# Multiple Attribute Group Decision-Making Methods Based on Trapezoidal Fuzzy Two-Dimensional Linguistic Partitioned Bonferroni Mean Aggregation Operators

**DOI:** 10.3390/ijerph15020194

**Published:** 2018-01-24

**Authors:** Kedong Yin, Benshuo Yang, Xuemei Li

**Affiliations:** 1 School of Economics, Ocean University of China, Qingdao 266100, China; yinkedong@ouc.edu.cn (K.Y.); qdybshuo@126.com (B.Y.); 2 Ocean Development Research Institute, Major Research Base of Humanities and Social Sciences of Ministry of Education, Ocean University of China, Qingdao 266100, China

**Keywords:** MAGDM, trapezoidal fuzzy two-dimensional linguistic information, partitioned Bonferroni mean aggregation operator

## Abstract

In this paper, we investigate multiple attribute group decision making (MAGDM) problems where decision makers represent their evaluation of alternatives by trapezoidal fuzzy two-dimensional uncertain linguistic variable. To begin with, we introduce the definition, properties, expectation, operational laws of trapezoidal fuzzy two-dimensional linguistic information. Then, to improve the accuracy of decision making in some case where there are a sort of interrelationship among the attributes, we analyze partition Bonferroni mean (PBM) operator in trapezoidal fuzzy two-dimensional variable environment and develop two operators: trapezoidal fuzzy two-dimensional linguistic partitioned Bonferroni mean (TF2DLPBM) aggregation operator and trapezoidal fuzzy two-dimensional linguistic weighted partitioned Bonferroni mean (TF2DLWPBM) aggregation operator. Furthermore, we develop a novel method to solve MAGDM problems based on TF2DLWPBM aggregation operator. Finally, a practical example is presented to illustrate the effectiveness of this method and analyses the impact of different parameters on the results of decision-making.

## 1. Introduction

Multiple attribute group decision making (MAGDM) is the process where the decision makers select the optimal alternative from all of the predefined alternatives by comparative analysis in terms of multiple attributes variables. MAGDM problems have successful applications in the management, scientific, political, cultural and other fields. In the fact decision-making process, decision makers are often trapped in using real number to evaluate alternatives, because the objective things are difficult to describe, and people’s judgments are subjective and uncertain. For example, elements like appearance, quality, portability and system fluency are taken into consideration when we determined to buy a laptop. Words like “convenient”, “general” and “inconvenient” are used to justify the portability of laptops, while in description of the system fluency, “fast” and “slow” are selected [1,2,3,4,5,6,7]. Zadeh [8,9,10,11] proposed the fuzzy set theory, which is a basis of the development of fuzzy multiple-attribute decision-making methods. Then, fuzzy set theory has rapid development and wide application in MADM and MAGDM problems [12,13,14,15,16]. For the sake of improving the accuracy of evaluation results, decision makers not only assess each alternatives from all attribute variables, but also demonstrate the reliability and stability of their evaluation. For example, in the process of evaluation on the rewards for the science and technology, experts need to analyze the reliability of the evaluation result. In this case, traditional one-dimensional linguistic information can be difficult to express both the evaluation results and the reliability of the evaluation simultaneously. Zhu et al. [17] proposed the 2-dimensional linguistic information to express decision opinions, which can handle more linguistic information than traditional fuzzy linguistic methods. The 2-dimensional linguistic information can be divided into two classes, the class I of the linguistic information is used to express the subjective evaluation of each alternative, and the class II is used for describing the reliability of result of the class I. This can subdivide the uncertainty in the decision-making process into the uncertainty of decision-making and the uncertainty of subjective cognition, which is helpful to improve the accuracy of the description of linguistic information for decision makers.

In recent years, studies on 2-dimensional linguistic information has been developed rapidly. Zhu et al. [17] used subjective judgement and reliability evaluation to describe 2-dimensional linguistic information and proposed an approach solved the assembly in complex conflict situations by using 2-dimensional linguistic information. However, the approach is difficult to solve the MADM problems, and the sequences of operation is more complex. Liu and Zhang [18] extended 2-dimensional linguistic information to 2-dimensional uncertain linguistic information, which can increase the range of 2-dimensional linguistic information and improve the accuracy of the description of linguistic information, and proposed an approach used the form of 2-dimensional uncertain linguistic information to solve the MAGDM problems. Zhang et al. [19] proposed evidence reasoning theory and built the second–dimensional semantic recognition framework, which reflects the evaluation information and behavior characteristics of decision-making problems. Yu et al. [20] showed that using 2-dimensional linguistic information in multiple decision making can avoid biased results by comparing 2-dimensional linguistic information and common linguistic information, and transformed linguistic information into the generalized triangle fuzzy number, which provided a new idea in the form of 2-dimensional linguistic information. Li et al. [21] proposed trapezoidal fuzzy 2-dimensional linguistic information in which class I information expressed by trapezoidal fuzzy number, and proposed the trapezoidal fuzzy two-dimensional linguistic power generalized aggregation (TF2DLPGA) and the trapezoidal fuzzy two-dimensional linguistic power generalized weighted aggregation (TF2DLPGWA) operators, then a multiple attribute decision method was developed. Liu et al. [22,23,24] introduced some operators under the 2-dimensional uncertain linguistic environment for solving MAGDM problems.

In real decision-making process, there exists some interrelationship among the attributes. The Bonferroni mean (BM), introduced by Bonferroni [25], establishes a conjunction among any pair of attributes and analyzes the interrelationship among them to evaluate each alternative. Based on Bonferroni mean operator, Yager [26,27,28] provided an interpretation of BM and suggested generalizations by transforming simple averaging into other mean type operators to enhance BM’s modeling capability. Up to now, more and more studies focus on applying BM operator to different decision fields or decision environments [29,30,31,32,33,34]. The BM operator can successfully solve the decision-making problem that each attribute variable have relationship with rest of attribute variables. However, in the real-life decision-making process, not all attribute variable have interrelationship with others. For example, taking into account a car selection problem, where choose the most appropriate car from numbers of car options based on four attributes: physical characteristics, power performance, technical features and customer excitement. It is found that the physical characteristic attribute is interrelated to the attributes power performance and technical features, however, there is no relationship between the physical characteristic attribute and the customer excitement attribute. In order to expand the application range of the BM operator, the partitioned BM (PBM) operator was proposed by Dutta [29], which has the capability to capture inter-relationship among the attributes with the assumption that attributes are partitioned into several unrelated classes and each attribute only has interrelationships with rest of the attributes in the same class. Then Dutta analyzed the linguistic weighted 2-tuple linguistic PBM (LW-2TLPBM) aggregation operator and proposed a method to solve MAGDM problems. Liu et al. [35] extended PBM to intuitionistic fuzzy sets (IFSs) and intuitionistic fuzzy numbers (IFNs), then proposed the intuitionistic fuzzy interaction partitioned Bonferroni mean (IFIPBM) and the intuitionistic fuzzy weighted interaction partitioned Bonferroni mean (IFWIPBM) operators. Liu et al. [36] proposed intuitionistic uncertain linguistic partitioned geometric Bonferroni mean (IULPBM) and its weighted form (WIULPBM) operators and developed an approach for solving the MAGDM problems under the intuitionistic uncertain linguistic environment.

In this paper, we combine trapezoidal fuzzy 2-dimensional linguistic information with a partitioned BM operator, and then, we propose trapezoidal fuzzy two-dimensional linguistic partitioned Bonferroni mean (TF2DLPBM) and trapezoidal fuzzy two-dimensional linguistic weighted partitioned Bonferroni mean (TF2DLWPBM) aggregation operators for solving MAGDM problems. Compared with traditional 2-dimensional linguistic information, class I information in trapezoidal fuzzy 2-dimensional linguistic information can be expressed by trapezoidal fuzzy number to increase the application range of the operator, which is more reasonable. The TF2DLPBM operator and the TF2DLWPBM operator have the capability to capture inter-relationship among the attributes with the preparatory work that attributes are partitioned into several unrelated class and each attribute only have interrelationship with rest attributes in the same class. In addition, decision makers can adjust the parameters according to their preferences and achieve the evaluation results of different preferences.

The remainder of this paper is arranged as follows: Section 2 introduces the concepts, characteristics, expectation, operational laws and distance measure of trapezoidal fuzzy numbers, two-dimension linguistic information and partitioned Bonferroni mean briefly. In Section 3, we propose the TF2DLPBM and TF2DLWPBM operators, then we give the definition and characteristics about them. An approach for solving MAGDM problems based on the TF2DLWPBM operator is proposed in Section 4. In Section 5, we give a practical example to explain and analysis our method, and compare it with the prominent existing methods. Finally, we discuss the conclusion in Section 6.

## 2. Preliminaries

### 2.1. The Trapezoidal Fuzzy Numbers

**Definition** **1.** *A trapezoidal fuzzy number* $\tilde{\alpha }$ *is defined as* $\tilde{\alpha }=\left(\alpha^{L},\alpha^{ML},\alpha^{MR},\alpha^{R}\right)$ *which satisfies the condition* $\alpha^{L}\leq \alpha^{ML}\leq \alpha^{MR}\leq \alpha^{R}$*, and its subordinate function a(x)*: *R*→[0, 1] *can be calculated as follows* [37,38]*:*

(1) $$a(x)=\{\begin{array}{l} \frac{x-\alpha^{L}}{\alpha^{ML}-\alpha^{L}},\;\;x\in [\alpha^{L},\alpha^{ML})\\ \;1,\;\;\;x\in [\alpha^{ML},\alpha^{MR})\\ \frac{x-\alpha^{R}}{\alpha^{MR}-\alpha^{R}},\;\;x\in [\alpha^{MR},\alpha^{R}]\\ \begin{array}{cc} 0, & x\in (-\infty ,\alpha^{L})\cup (\alpha^{R},+\infty ) \end{array} \end{array}$$

*where the any element x of the subordinate function is real number and subordinate function* a(x) *is a regular, consecutive convex function, which exhibits the membership of the element* x *to the set* $\tilde{a}$*, Specially, trapezoidal fuzzy number can be transformed into triangular fuzzy number or crisp number when* $\alpha^{L}\leq \alpha^{ML}\leq \alpha^{MR}\leq \alpha^{R}$ *or* $\alpha^{L}=\alpha^{ML}=\alpha^{MR}=\alpha^{R}$*.*

Suppose $\tilde{\alpha }=\left(\alpha^{L},\alpha^{ML},\alpha^{MR},\alpha^{R}\right),$$\tilde{\beta }=\left(\beta^{L},\beta^{ML},\beta^{MR},\beta^{R}\right)$ are any two trapezoidal fuzzy numbers, and $\alpha^{L},\alpha^{ML},\alpha^{MR},\alpha^{R},\beta^{L},\beta^{ML},\beta^{MR},\beta^{R}$ are real numbers, then the trapezoidal fuzzy numbers operational rules are indicated as follows:

(2) $$\tilde{\alpha }+\tilde{\beta }=(\alpha^{L}+\beta^{L},\alpha^{ML}+\beta^{ML},\alpha^{MR}+\beta^{MR},\alpha^{R}+\beta^{R})$$

(3) $$\tilde{\alpha }-\tilde{\beta }=(\alpha^{L}-\beta^{R},\alpha^{ML}-\beta^{MR},\alpha^{MR}-\beta^{ML},\alpha^{R}-\beta^{L})$$

(4) $$\tilde{\alpha }\tilde{\beta }=(\alpha^{L}\beta^{L},\alpha^{ML}\beta^{ML},\alpha^{MR}\beta^{MR},\alpha^{R}\beta^{R})$$

(5) $$\tilde{\alpha }/\tilde{\beta }=(\alpha^{L}/\beta^{R},\alpha^{ML}/\beta^{MR},\alpha^{MR}/\beta^{ML},\alpha^{R}/\beta^{L})$$

(6) $$\lambda \tilde{\alpha }=(\lambda \alpha^{L},\lambda \alpha^{ML},\lambda \alpha^{MR},\lambda \alpha^{R}),\lambda \geq 0$$

(7) $${\left(\tilde{\alpha }\right)}^{r}=({\left(\alpha^{L}\right)}^{r},{\left(\alpha^{ML}\right)}^{r},{\left(\alpha^{MR}\right)}^{r},{\left(\alpha^{R}\right)}^{r}),r>0$$

The distance between $\tilde{\alpha }=\left(\alpha^{L},\alpha^{ML},\alpha^{MR},\alpha^{R}\right)$ and $\tilde{\beta }=\left(\beta^{L},\beta^{ML},\beta^{MR},\beta^{R}\right)$ is defined as follows:

(8) $$d(\tilde{\alpha },\tilde{\beta })=\sqrt{\frac{{\left(\alpha^{L}-\beta^{L}\right)}^{2}+{\left(\alpha^{ML}-\beta^{ML}\right)}^{2}+{\left(\alpha^{MR}-\beta^{MR}\right)}^{2}+{\left(\alpha^{R}-\beta^{R}\right)}^{2}}{4}}$$

### 2.2. The Linguistic Set

Let $S=\left(s_{0},s_{1},\ldots ,s_{l-1}\right)$ be linguistic term set consists of finite and odd number of elements, which means l is an odd value. Generally, l can be set to 3, 5, 7, 9, etc. For instance, when *l* = 5 $S=\left(s_{0},s_{1},s_{2},s_{3},s_{4}\right)$ = {poor, slightly-poor, fair, slightly-good, good}. Here, $s_{\alpha }$, α=0,1,…,l−1 can be called a original linguistic variable [39].

Suppose $s_{i}$ and $s_{j}$ are any two elements in linguistic term set S, the conditions they need to meet are as follows [40,41]:

1. If *i* > *j*, then *s_i_* > *s_j_* (that means $s_{i}$ is better than $s_{j}$);
2. there exists negative operator: *neg*(*s_i_*) > *s_l_*_−*i*−1_;
3. if *s_i_* ≥ *s_j_*, ($s_{i}$ is not worse than $s_{j}$), then max(*s_i_,s_j_*) = *s_i_*;
4. if $s_{i}\leq s_{j}$, ($s_{i}$ is not better than $s_{j}$), then min(*s_i_,s_j_*) = *s_i_*.

### 2.3. The Trapezoidal Fuzzy Two-Dimensional Linguistic Variable

**Definition** **2** [20,21]**.** *Let* $\hat{s}=\left(\left[a,b,c,d\right],s_{\theta }\right)$ *where* [a,b,c,d] *is a trapezoidal fuzzy number and* $s_{\theta }$ *is linguistic information, all of them are essential in a trapezoidal fuzzy two-dimensional linguistic variable because decision makers can use the first part to describe the assessment value of the evaluated object, and the second part to estimate the reliability of the first part. Then* $\hat{s}$ *is called the trapezoidal fuzzy two-dimensional linguistic variable.*

Suppose ${\hat{s}}_{1}=\left(\left[a_{1},b_{1},c_{1},d_{1}\right],s_{\theta_{1}}\right)$ and ${\hat{s}}_{2}=\left(\left[a_{2},b_{2},c_{2},d_{2}\right],s_{\theta_{2}}\right)$ be any two trapezoidal fuzzy two-dimensional linguistic variables, and $a_{1},b_{1},c_{1},d_{1},a_{2},b_{2},c_{2},d_{2}\geq 0$, then the operational laws are defined as follows:

(9) $${\hat{s}}_{1}⊕{\hat{s}}_{2}=([a_{1}+a_{2},b_{1}+b_{2},c_{1}+c_{2},d_{1}+d_{2}],s_{\min(\theta_{1},\theta_{2})})$$

(10) $${\hat{s}}_{1}⊗{\hat{s}}_{2}=([a_{1}a_{2},b_{1}b_{2},c_{1}c_{2},d_{1}d_{2}],s_{\min(\theta_{1},\theta_{2})})$$

(11) $${\hat{s}}_{1}/{\hat{s}}_{2}=([a_{1}/d_{2},b_{1}/c_{2},c_{1}/b_{2},d_{1}/a_{2}],s_{\min(\theta_{1},\theta_{2})})\;\mathrm{where}\;a_{2},b_{2},c_{2},d_{2}\neq 0$$

(12) $$\lambda {\hat{s}}_{1}=([\lambda a_{1},\lambda b_{1},\lambda c_{1},\lambda d_{1}],s_{\theta_{1}}),\lambda \geq 0$$

(13) $${\left({\hat{s}}_{1}\right)}^{\lambda }=([a_{1}{}^{\lambda },b_{1}{}^{\lambda },c_{1}{}^{\lambda },d_{1}{}^{\lambda }],s_{\theta_{1}}),\lambda >0$$

**Definition** **3** [21]**.** *Let* $\hat{s}=\left(\left[a,b,c,d\right],s_{\theta }\right)$ *be a trapezoidal fuzzy two-dimensional linguistic variable, then the expectation of trapezoidal fuzzy two-dimensional linguistic variable is defined as below:*

(14) $$E(\hat{s})=\frac{a+b+c+d}{4}\times \frac{\theta }{l-1}$$

*Let* ${\hat{s}}_{1}=\left(\left[a_{1},b_{1},c_{1},d_{1}\right],s_{\theta_{1}}\right)$ and ${\hat{s}}_{2}=\left(\left[a_{2},b_{2},c_{2},d_{2}\right],s_{\theta_{2}}\right)$ *be any two trapezoidal fuzzy two-dimensional linguistic variables, we can compare* ${\hat{s}}_{1}$ *and* ${\hat{s}}_{2}$ *by using the expectation of them, if* $E\left({\hat{s}}_{1}\right)\geq E\left({\hat{s}}_{2}\right)$ *then* ${\hat{s}}_{1}\geq {\hat{s}}_{2}$*, or vice versa*.

### 2.4. Partitioned Bonferroni Mean

**Definition** **4** [25]**.** *For any p,q ≥* 0 *with p+q ≥* 0, *the BM aggregation operator of dimension* n *is a mapping BM:* (*R*^+^)*^n^*→*R*^+^ *such that:*

(15) $${\mathrm{BM}}^{p,q}(a_{1},a_{2},\cdots ,a_{n})={\left(\frac{1}{n(n-1)}\sum_{\begin{array}{c} i,j=1\\ i\neq j \end{array}}^{n}a_{i}^{p}a_{j}^{q}\right)}^{\frac{1}{p+q}}$$

*where* $R^{+}$ *is the set of non-negative real number. BM operator was widely applied in the multiple attribute decision making problem with the assumption that each attribute is related to the rest of the attributes.*

**Definition** **5** [29,42]**.** *For any p,q ≥ 0 with p+q ≥ 0, the PBM operator is a mapping PBM:* [0, 1]*^n^→*[0.1] *such that:*

(16) $$\mathrm{PBM}(a_{1},a_{2},\cdots ,a_{n})=\frac{1}{e}\left(\sum_{h=1}^{e}{\left(\frac{1}{\left|P_{h}\right|}\sum_{i\in P_{h}}a_{i}^{p}\left(\frac{1}{|P_{h}|-1}\sum_{\begin{array}{c} j\in P_{h}\\ j\neq i \end{array}}a_{j}^{q}\right)\right)}^{\frac{1}{p+q}}\right)$$

*Let attribute set* $C=\left(A_{1},A_{2},\ldots ,A_{n}\right)$ *consist of the sets of inputs* $A=\left(a_{1},a_{2},\ldots ,a_{n}\right)$*, which have a relationship with the attribute set. Fundamentally, a_i_ is a non-negative real number. Divide attribute set C into e distinct classes P*_1_, *P*_2_,…*P_e_ such that* $P_{i}\cap^{\text{​}}P_{j}=\emptyset$*, and* $\overset{e}{\underset{h=1}{\cup }}P_{h}=C$ *based on an interrelationship pattern. Assume that attributes of each P_i_ are interrelated to each other and there is no relationship with other attributes in other classes. In Equation* (16) $\sum_{P_{h}}a_{i}{}^{p}\left[\left(1/\left|P_{h}-1\right|\right)\sum_{j\in P_{h},j\neq 1}a_{j}{}^{q}\right]$ *shows the satisfaction of the attribute a_i_* *with the average satisfaction of the attributes belong to P_h_* *except a_i_*.

Some important theorems resulting from Equation (16) are shown as below:

**Theorem** **1.** *(Idempotency) Let p,q ≥* 0 *and a_i_ = a, for all* i=1,2,…,n*. Then*

(17) PBM(a,a,⋯,a)=a

**Theorem** **2.** *(Monotonicity) Let p,q ≥* 0 *and a_i_* *≤ b_i_, for all* i=1,2,…,n*. Then*

(18) $$\mathrm{PBM}(a_{1},a_{2},\cdots ,a_{n})\leq \mathrm{PBM}(b_{1},b_{2},\cdots ,b_{n})$$

**Theorem** **3.** *(Boundedness) Let* $a_{l}=\min_{i}a_{i\;}$ *and* $a_{u}=\max_{i}a_{i}$*, then, for any p,q ≥* 0

(19) $$a_{l}\leq \mathrm{PBM}(a_{1},a_{2},\cdots ,a_{n})\leq a_{u}$$

## 3. The Trapezoidal Fuzzy Two-Dimensional Linguistic Partitioned Bonferroni Mean Aggregation Operators

### 3.1. The Trapezoidal Fuzzy Two-Dimensional Linguistic Partitioned Bonferroni Mean Aggregation Operators

In real decision-making problem, each attribute may be interrelated to some attributes and not be interrelated to the other attributes. To improve the accuracy of decision making, we partition the attribute set into several classes and ensure that all attributes of each class have an interrelationship with other attributes in the same class and have no interrelationship among attributes from other classes. So as to fully consider the interrelationship among the trapezoidal fuzzy two-dimensional linguistic variables, we propose the TF2DLPBM aggregation operator which can be defined as follows:

**Definition** **6.** *Let* ${\hat{s}}_{j}=\left(\left[a_{j},b_{j},c_{j},d_{j}\right],s_{\theta_{j}}\right)$ *(j* = 1,2,…,*n*) *be a trapezoidal fuzzy two-dimensional linguistic variable, and the trapezoidal fuzzy two-dimensional linguistic partitioned Bonferroni mean (TF2DLPBM):* $\Omega^{n}\to \Omega$*, if*

(20) $$\mathrm{TF}2\mathrm{DLPBM}({\hat{s}}_{1},{\hat{s}}_{2},\cdots ,{\hat{s}}_{n})=\frac{1}{e}\left(\sum_{h=1}^{e}{\left(\frac{1}{\left|P_{h}\right|}\sum_{i\in P_{h}}{\hat{s}}_{i}^{p}\left(\frac{1}{|P_{h}|-1}\sum_{\begin{array}{c} j\in P_{h}\\ j\neq i \end{array}}{\hat{s}}_{j}^{q}\right)\right)}^{\frac{1}{p+q}}\right)$$

*where* Ω *is the set of all trapezoidal fuzzy two-dimensional linguistic variables, p and q are parameters such that p* ∈ (0,∞) *and q* ∈ (0,∞)*,* $\left|P_{h}\right|$ *denotes the cardinality of P_h_.*

**Theorem** **4.** *Let* ${\hat{s}}_{j}=\left(\left[a_{j},b_{j},c_{j},d_{j}\right],s_{\theta_{j}}\right)$ *(j* = 1,2,…,*n*) *be a collection of the trapezoidal fuzzy two-dimensional linguistic variables, then the result aggregated from Definition 6 is still a trapezoidal fuzzy two-dimensional linguistic variable, and also*

(21) $$\mathrm{TF}2\mathrm{DLPBM}({\hat{s}}_{1},{\hat{s}}_{2},\cdots ,{\hat{s}}_{n})=([\frac{1}{e}\left(\sum_{h=1}^{e}{\left(\frac{1}{\left|P_{h}\right|}\sum_{i\in P_{h}}a_{i}^{p}\left(\frac{1}{|P_{h}|-1}\sum_{\begin{array}{c} j\in P_{h}\\ j\neq i \end{array}}a_{j}^{q}\right)\right)}^{\frac{1}{p+q}}\right),\;\frac{1}{e}\left(\sum_{h=1}^{e}{\left(\frac{1}{\left|P_{h}\right|}\sum_{i\in P_{h}}b_{i}^{p}\left(\frac{1}{|P_{h}|-1}\sum_{\begin{array}{c} j\in P_{h}\\ j\neq i \end{array}}b_{j}^{q}\right)\right)}^{\frac{1}{p+q}}\right),\frac{1}{e}\left(\sum_{h=1}^{e}{\left(\frac{1}{\left|P_{h}\right|}\sum_{i\in P_{h}}c_{i}^{p}\left(\frac{1}{|P_{h}|-1}\sum_{\begin{array}{c} j\in P_{h}\\ j\neq i \end{array}}c_{j}^{q}\right)\right)}^{\frac{1}{p+q}}\right),\;\frac{1}{e}\left(\sum_{h=1}^{e}{\left(\frac{1}{\left|P_{h}\right|}\sum_{i\in P_{h}}d_{i}^{p}\left(\frac{1}{|P_{h}|-1}\sum_{\begin{array}{c} j\in P_{h}\\ j\neq i \end{array}}d_{j}^{q}\right)\right)}^{\frac{1}{p+q}}\right)],s_{\underset{\begin{array}{c} i,j\\ i\neq j \end{array}}{\min}(\theta_{i},\theta_{j})})$$

**Proof.**  According to the operational rules of the trapezoidal fuzzy two-dimensional linguistic variables, we have:

$${\hat{s}}_{i}{}^{p}=([(a_{i}{}^{p}),(b_{i}{}^{p}),(c_{i}{}^{p}),(d_{i}{}^{p})],s_{\underset{i}{\min}\theta_{i}}),\;\;{\hat{s}}_{j}{}^{q}=([(a_{j}{}^{q}),(b_{j}{}^{q}),(c_{j}{}^{q}),(d_{j}{}^{q})],s_{\underset{j}{\min}\theta_{j}})$$

and:

$$\frac{1}{|P_{h}|-1}\sum_{\begin{array}{c} j\in P_{h}\\ j\neq i \end{array}}{\hat{s}}_{j}^{q}=([\left(\frac{1}{|P_{h}|-1}\sum_{\begin{array}{c} j\in P_{h}\\ j\neq i \end{array}}a_{j}^{q}\right),\left(\frac{1}{|P_{h}|-1}\sum_{\begin{array}{c} j\in P_{h}\\ j\neq i \end{array}}b_{j}^{q}\right),\left(\frac{1}{|P_{h}|-1}\sum_{\begin{array}{c} j\in P_{h}\\ j\neq i \end{array}}c_{j}^{q}\right),\left(\frac{1}{|P_{h}|-1}\sum_{\begin{array}{c} j\in P_{h}\\ j\neq i \end{array}}d_{j}^{q}\right),s_{\underset{\begin{array}{c} j \end{array}}{\min}\theta_{j}})$$

then:

$$\frac{1}{\left|P_{h}\right|}\sum_{i\in P_{h}}{\hat{s}}_{i}^{p}\left(\frac{1}{|P_{h}|-1}\sum_{\begin{array}{c} j\in P_{h}\\ j\neq i \end{array}}{\hat{s}}_{j}^{q}\right)=([\frac{1}{\left|P_{h}\right|}\sum_{i\in P_{h}}a_{i}^{p}\left(\frac{1}{|P_{h}|-1}\sum_{\begin{array}{c} j\in P_{h}\\ j\neq i \end{array}}a_{j}^{q}\right),\;\frac{1}{\left|P_{h}\right|}\sum_{i\in P_{h}}b_{i}^{p}\left(\frac{1}{|P_{h}|-1}\sum_{\begin{array}{c} j\in P_{h}\\ j\neq i \end{array}}b_{j}^{q}\right),\frac{1}{\left|P_{h}\right|}\sum_{i\in P_{h}}c_{i}^{p}\left(\frac{1}{|P_{h}|-1}\sum_{\begin{array}{c} j\in P_{h}\\ j\neq i \end{array}}c_{j}^{q}\right),\;\frac{1}{\left|P_{h}\right|}\sum_{i\in P_{h}}d_{i}^{p}\left(\frac{1}{|P_{h}|-1}\sum_{\begin{array}{c} j\in P_{h}\\ j\neq i \end{array}}d_{j}^{q}\right)],s_{\underset{\begin{array}{c} i,j\\ i\neq j \end{array}}{\min}(\theta_{i},\theta_{j})})$$

and:

$$\frac{1}{e}\left(\sum_{h=1}^{e}{\left(\frac{1}{\left|P_{h}\right|}\sum_{i\in P_{h}}{\hat{s}}_{i}^{p}\left(\frac{1}{|P_{h}|-1}\sum_{\begin{array}{c} j\in P_{h}\\ j\neq i \end{array}}{\hat{s}}_{j}^{q}\right)\right)}^{\frac{1}{p+q}}\right)=([\frac{1}{e}\left(\sum_{h=1}^{e}{\left(\frac{1}{\left|P_{h}\right|}\sum_{i\in P_{h}}a_{i}^{p}\left(\frac{1}{|P_{h}|-1}\sum_{\begin{array}{c} j\in P_{h}\\ j\neq i \end{array}}a_{j}^{q}\right)\right)}^{\frac{1}{p+q}}\right),\;\frac{1}{e}\left(\sum_{h=1}^{e}{\left(\frac{1}{\left|P_{h}\right|}\sum_{i\in P_{h}}b_{i}^{p}\left(\frac{1}{|P_{h}|-1}\sum_{\begin{array}{c} j\in P_{h}\\ j\neq i \end{array}}b_{j}^{q}\right)\right)}^{\frac{1}{p+q}}\right),\frac{1}{e}\left(\sum_{h=1}^{e}{\left(\frac{1}{\left|P_{h}\right|}\sum_{i\in P_{h}}c_{i}^{p}\left(\frac{1}{|P_{h}|-1}\sum_{\begin{array}{c} j\in P_{h}\\ j\neq i \end{array}}c_{j}^{q}\right)\right)}^{\frac{1}{p+q}}\right),\;\frac{1}{e}\left(\sum_{h=1}^{e}{\left(\frac{1}{\left|P_{h}\right|}\sum_{i\in P_{h}}d_{i}^{p}\left(\frac{1}{|P_{h}|-1}\sum_{\begin{array}{c} j\in P_{h}\\ j\neq i \end{array}}d_{j}^{q}\right)\right)}^{\frac{1}{p+q}}\right)],s_{\underset{\begin{array}{c} i,j\\ i\neq j \end{array}}{\min}(\theta_{i},\theta_{j})})$$

which completes the proof. □

The TF2DLPBM aggregation operator has the following properties.

**Theorem** **5.** *(Commutativity) Let* $\left({{\hat{s}}^{\prime }}_{1},{{\hat{s}}^{\prime }}_{2},\cdots ,{{\hat{s}}^{\prime }}_{n}\right)$ *be any permutation of* $\left({\hat{s}}_{1},{\hat{s}}_{2},\cdots ,{\hat{s}}_{n}\right)$*, then*

(22) $$\mathrm{TF}2\mathrm{DLPBM}({{\hat{s}}^{\prime }}_{1},{{\hat{s}}^{\prime }}_{2},\cdots ,{{\hat{s}}^{\prime }}_{n})=\mathrm{TF}2\mathrm{DLPBM}({\hat{s}}_{1},{\hat{s}}_{2},\cdots ,{\hat{s}}_{n})$$

**Proof.**  Let

$$\mathrm{TF}2\mathrm{DLPBM}({{\hat{s}}^{\prime }}_{1},{{\hat{s}}^{\prime }}_{2},\cdots ,{{\hat{s}}^{\prime }}_{n})=\frac{1}{e}\left(\sum_{h=1}^{e}{\left(\frac{1}{\left|P_{h}\right|}\sum_{i\in P_{h}}{{\hat{s}}^{\prime }}_{i}^{p}\left(\frac{1}{|P_{h}|-1}\sum_{\begin{array}{c} j\in P_{h}\\ j\neq i \end{array}}{{\hat{s}}^{\prime }}_{j}^{q}\right)\right)}^{\frac{1}{p+q}}\right)$$

$$\mathrm{TF}2\mathrm{DLPBM}({\hat{s}}_{1},{\hat{s}}_{2},\cdots ,{\hat{s}}_{n})=\frac{1}{e}\left(\sum_{h=1}^{e}{\left(\frac{1}{\left|P_{h}\right|}\sum_{i\in P_{h}}{\hat{s}}_{i}^{p}\left(\frac{1}{|P_{h}|-1}\sum_{\begin{array}{c} j\in P_{h}\\ j\neq i \end{array}}{\hat{s}}_{j}^{q}\right)\right)}^{\frac{1}{p+q}}\right)$$

Since $\left({{\hat{s}}^{\prime }}_{1},{{\hat{s}}^{\prime }}_{2},\cdots ,{{\hat{s}}^{\prime }}_{n}\right)$ is any permutation of $\left({\hat{s}}_{1},{\hat{s}}_{2},\cdots ,{\hat{s}}_{n}\right)$, we have:

$$\frac{1}{|P_{h}|-1}\sum_{\begin{array}{c} j\in P_{h}\\ j\neq i \end{array}}{\hat{s}}_{j}^{q}=\frac{1}{|P_{h}|-1}\sum_{\begin{array}{c} j\in P_{h}\\ j\neq i \end{array}}{\hat{s}}_{j}^{q}$$

$$\frac{1}{\left|P_{h}\right|}\sum_{i\in P_{h}}{\hat{s}}_{i}^{p}\left(\frac{1}{|P_{h}|-1}\sum_{\begin{array}{c} j\in P_{h}\\ j\neq i \end{array}}{\hat{s}}_{j}^{q}\right)=\frac{1}{\left|P_{h}\right|}\sum_{i\in P_{h}}{\hat{s}}_{i}^{p}\left(\frac{1}{|P_{h}|-1}\sum_{\begin{array}{c} j\in P_{h}\\ j\neq i \end{array}}{\hat{s}}_{j}^{q}\right)$$

Thus:

$$\mathrm{TF}2\mathrm{DLPBM}({{\hat{s}}^{\prime }}_{1},{{\hat{s}}^{\prime }}_{2},\cdots ,{{\hat{s}}^{\prime }}_{n})=\mathrm{TF}2\mathrm{DLPBM}({\hat{s}}_{1},{\hat{s}}_{2},\cdots ,{\hat{s}}_{n})$$

□

**Theorem** **6.** *(Idempotency) Let* ${\hat{s}}_{j}=\hat{s},j=1,2,\cdots ,n$*, then:*

(23) $$\mathrm{TF}2\mathrm{DLPBM}({{\hat{s}}^{\prime }}_{1},{{\hat{s}}^{\prime }}_{2},\cdots ,{{\hat{s}}^{\prime }}_{n})=\hat{s}$$

**Proof.**  Since ${\hat{s}}_{j}=\hat{s}$, for all j, we have:

$$\mathrm{TF}2\mathrm{DLPBM}({\hat{s}}_{1},{\hat{s}}_{2},\cdots ,{\hat{s}}_{n})=\frac{1}{e}\left(\sum_{h=1}^{e}{\left(\frac{1}{\left|P_{h}\right|}\sum_{i\in P_{h}}{\hat{s}}_{}^{p}\left(\frac{1}{|P_{h}|-1}\sum_{\begin{array}{c} j\in P_{h}\\ j\neq i \end{array}}{\hat{s}}_{}^{q}\right)\right)}^{\frac{1}{p+q}}\right)\;=\frac{1}{e}\left(\sum_{h=1}^{e}{\left(\frac{1}{\left|P_{h}\right|}\cdot \sum_{i\in P_{h}}{\hat{s}}^{p+q}\right)}^{\frac{1}{p+q}}\right)=\frac{1}{e}\left(\sum_{h=1}^{e}{\left({\hat{s}}^{p+q}\right)}^{\frac{1}{p+q}}\right)=\frac{1}{e}\left(\sum_{h=1}^{e}\hat{s}\right)=\hat{s}$$

□

**Theorem** **7.** *(Boundedness) The TF2DLPBM operator lies between the max and min operators:*

(24) $$\min({\hat{s}}_{1},{\hat{s}}_{2},\cdots ,{\hat{s}}_{n})\leq \mathrm{TF}2\mathrm{DLPBM}({\hat{s}}_{1},{\hat{s}}_{2},\cdots ,{\hat{s}}_{n})\leq \max({\hat{s}}_{1},{\hat{s}}_{2},\cdots ,{\hat{s}}_{n})$$

**Proof.**  Let $\hat{a}=\min\left({\hat{s}}_{1},{\hat{s}}_{2},\ldots ,{\hat{s}}_{n}\right)$, $\hat{b}=\max\left({\hat{s}}_{1},{\hat{s}}_{2},\ldots ,{\hat{s}}_{n}\right)$

Since $\hat{a}\leq {\hat{s}}_{j}\leq \hat{b}$, then:

$$\frac{1}{e}\left(\sum_{h=1}^{e}{\left(\frac{1}{\left|P_{h}\right|}\sum_{i\in P_{h}}{\hat{a}}_{}^{p}\left(\frac{1}{|P_{h}|-1}\sum_{\begin{array}{c} j\in P_{h}\\ j\neq i \end{array}}{\hat{a}}_{}^{q}\right)\right)}^{\frac{1}{p+q}}\right)\leq \frac{1}{e}\left(\sum_{h=1}^{e}{\left(\frac{1}{\left|P_{h}\right|}\sum_{i\in P_{h}}{\hat{s}}_{}^{p}\left(\frac{1}{|P_{h}|-1}\sum_{\begin{array}{c} j\in P_{h}\\ j\neq i \end{array}}{\hat{s}}_{}^{q}\right)\right)}^{\frac{1}{p+q}}\right)\leq \frac{1}{e}\left(\sum_{h=1}^{e}{\left(\frac{1}{\left|P_{h}\right|}\sum_{i\in P_{h}}{\hat{b}}_{}^{p}\left(\frac{1}{|P_{h}|-1}\sum_{\begin{array}{c} j\in P_{h}\\ j\neq i \end{array}}{\hat{b}}_{}^{q}\right)\right)}^{\frac{1}{p+q}}\right)$$

That is:

$$\hat{a}\leq \frac{1}{e}\left(\sum_{h=1}^{e}{\left(\frac{1}{\left|P_{h}\right|}\sum_{i\in P_{h}}{\hat{s}}_{}^{p}\left(\frac{1}{|P_{h}|-1}\sum_{\begin{array}{c} j\in P_{h}\\ j\neq i \end{array}}{\hat{s}}_{}^{q}\right)\right)}^{\frac{1}{p+q}}\right)\leq \hat{b}$$

$$\min({\hat{s}}_{1},{\hat{s}}_{2},\cdots ,{\hat{s}}_{n})\leq \mathrm{TF}2\mathrm{DLPBM}({\hat{s}}_{1},{\hat{s}}_{2},\cdots ,{\hat{s}}_{n})\leq \max({\hat{s}}_{1},{\hat{s}}_{2},\cdots ,{\hat{s}}_{n})$$

□

In the following, we will discuss some cases of the TTFLPBM operator

1. When *q* = 0:
  (25) $${\mathrm{TF}2\mathrm{DLPBM}}^{p,0}({\hat{s}}_{1},{\hat{s}}_{2},\cdots ,{\hat{s}}_{n})=\frac{1}{e}\left({\sum_{h=1}^{e}\left(\frac{1}{\left|P_{h}\right|}\sum_{i\in P_{h}}{\hat{s}}_{i}^{p}\right)}^{\frac{p}{1}}\right)\;=([\frac{1}{e}\left(\sum_{h=1}^{e}{\left(\frac{1}{\left|P_{h}\right|}\sum_{i\in P_{h}}a_{i}^{p}\right)}^{\frac{1}{p}}\right),\frac{1}{e}\left(\sum_{h=1}^{e}{\left(\frac{1}{\left|P_{h}\right|}\sum_{i\in P_{h}}b_{i}^{p}\right)}^{\frac{1}{p}}\right),\frac{1}{e}\left(\sum_{h=1}^{e}{\left(\frac{1}{\left|P_{h}\right|}\sum_{i\in P_{h}}c_{i}^{p}\right)}^{\frac{1}{p}}\right),\;\frac{1}{e}\left(\sum_{h=1}^{e}{\left(\frac{1}{\left|P_{h}\right|}\sum_{i\in P_{h}}d_{i}^{p}\right)}^{\frac{1}{p}}\right)],s_{\underset{\begin{array}{c} i \end{array}}{\min}\theta_{i}})$$
2. When *p* = 1, *q* = 0:
  (26) $${\mathrm{TF}2\mathrm{DLPBM}}^{1,0}({\hat{s}}_{1},{\hat{s}}_{2},\cdots ,{\hat{s}}_{n})=\frac{1}{e}\left(\sum_{h=1}^{e}\left(\frac{1}{\left|P_{h}\right|}\sum_{i\in P_{h}}{\hat{s}}_{i}^{}\right)\right)\;=([\frac{1}{e}\sum_{h=1}^{e}\left(\frac{1}{\left|P_{h}\right|}\sum_{i\in P_{h}}a_{i}^{}\right),\frac{1}{e}\sum_{h=1}^{e}\left(\frac{1}{\left|P_{h}\right|}\sum_{i\in P_{h}}b_{i}^{}\right),\frac{1}{e}\sum_{h=1}^{e}\left(\frac{1}{\left|P_{h}\right|}\sum_{i\in P_{h}}c_{i}^{}\right),\;\frac{1}{e}\sum_{h=1}^{e}\left(\frac{1}{\left|P_{h}\right|}\sum_{i\in P_{h}}d_{i}^{}\right)],s_{\underset{\begin{array}{c} i \end{array}}{\min}\theta_{i}})$$
3. When *p* = 1, *q* = 1
  (27) $${\mathrm{TF}2\mathrm{DLPBM}}^{1,1}({\hat{s}}_{1},{\hat{s}}_{2},\cdots ,{\hat{s}}_{n})=\frac{1}{e}\left(\sum_{h=1}^{e}{\left(\frac{1}{\left|P_{h}\right|}\sum_{i\in P_{h}}{\hat{s}}_{i}^{}\left(\frac{1}{|P_{h}|-1}\sum_{\begin{array}{c} j\in P_{h}\\ j\neq i \end{array}}{\hat{s}}_{j}^{}\right)\right)}^{\frac{1}{2}}\right)=([\frac{1}{e}\left(\sum_{h=1}^{e}{\left(\frac{1}{\left|P_{h}\right|}\sum_{i\in P_{h}}a_{i}^{}\left(\frac{1}{|P_{h}|-1}\sum_{\begin{array}{c} j\in P_{h}\\ j\neq i \end{array}}a_{j}^{}\right)\right)}^{\frac{1}{2}}\right),\frac{1}{e}\left(\sum_{h=1}^{e}{\left(\frac{1}{\left|P_{h}\right|}\sum_{i\in P_{h}}b_{i}^{}\left(\frac{1}{|P_{h}|-1}\sum_{\begin{array}{c} j\in P_{h}\\ j\neq i \end{array}}b_{j}^{}\right)\right)}^{\frac{1}{2}}\right),\frac{1}{e}\left(\sum_{h=1}^{e}{\left(\frac{1}{\left|P_{h}\right|}\sum_{i\in P_{h}}c_{i}^{}\left(\frac{1}{|P_{h}|-1}\sum_{\begin{array}{c} j\in P_{h}\\ j\neq i \end{array}}c_{j}^{}\right)\right)}^{\frac{1}{2}}\right),\frac{1}{e}\left(\sum_{h=1}^{e}{\left(\frac{1}{\left|P_{h}\right|}\sum_{i\in P_{h}}d_{i}^{}\left(\frac{1}{|P_{h}|-1}\sum_{\begin{array}{c} j\in P_{h}\\ j\neq i \end{array}}d_{j}^{}\right)\right)}^{\frac{1}{2}}\right)],s_{\underset{\begin{array}{c} i,j\\ i\neq j \end{array}}{\min}(\theta_{i},\theta_{j})})$$

### 3.2. The Trapezoidal Fuzzy Two-Dimensional Linguistic Weighted Partitioned Bonferroni Mean Aggregation Operators

It is noteworthy that the TF2DLPBM does not take into account the importance of all the objects $\left({\hat{s}}_{1},{\hat{s}}_{2}\ldots .{\hat{s}}_{n}\right)$. However, in many cases, we must differentiate the each object on the basis of their degrees of importance. Thus, we give different weights to different objects and establish TF2DLWPM aggregation operator that consider weight vector of objects in this subsection.

**Definition** **7** *Let* ${\hat{s}}_{j}=\left(\left[a_{j},b_{j},c_{j},d_{j}\right],s_{\theta_{j}}\right)$ *(j* = 1,2,…,*n*) *be a collection of trapezoidal fuzzy two-dimensional linguistic variables, and the trapezoidal fuzzy two-dimensional linguistic weighted partitioned Bonferroni mean (TF2DLWPBM):* $\Omega^{n}\to \Omega$*, if*

(28) $$\mathrm{TF}2\mathrm{DLWPBM}({\hat{s}}_{1},{\hat{s}}_{2},\cdots ,{\hat{s}}_{n})=\frac{1}{e}\left(\sum_{h=1}^{e}{\left(\frac{1}{\left|P_{h}\right|}\sum_{i\in P_{h}}{\left(\omega_{i}{\hat{s}}_{i}\right)}^{p}\left(\frac{1}{|P_{h}|-1}\sum_{\begin{array}{c} j\in P_{h}\\ j\neq i \end{array}}{\left(\omega_{j}{\hat{s}}_{j}\right)}^{q}\right)\right)}^{\frac{1}{p+q}}\right)$$

*where* Ω *is the set of all trapezoidal fuzzy two-dimensional linguistic numbers, and ω_i_* (*i* = 1,2,…,*n*) *indicates the relative importance of the input argument and satisfies the conditions:* $\omega_{i}\geq 0,\;\sum_{i=1}^{n}\omega_{i}=1$*, p and q are parameters such that p* ∈ (0,∞) *and q* ∈ (0,∞)*. Then TF2DLWPBM called the trapezoidal fuzzy two-dimensional linguistic weighted partitioned Bonferroni mean aggregation operator.*

**Theorem** **8.** *Let* ${\hat{s}}_{i}=\left(\left[a_{i},b_{i},c_{i},d_{i}\right],s_{\theta_{i}}\right)$ *(i* = 1,2,…,*n*) *be a collection of the trapezoidal fuzzy two-dimensional linguistic variables, and* $\omega ={\left(\omega_{1},\omega_{2},\ldots ,\omega_{n}\right)}^{T}$ *is the weight vector of* ${\hat{s}}_{i}\left(i=1,2,\ldots ,n\right),$$\omega_{i}\geq 0,\;\sum_{i=1}^{n}\omega_{i}=1$*, then, the result is still a trapezoidal fuzzy two-dimensional linguistic variable, and also*

(29) $$\mathrm{TF}2\mathrm{DLWPBM}({\hat{s}}_{1},{\hat{s}}_{2},\cdots ,{\hat{s}}_{n})=([\frac{1}{e}\left(\sum_{h=1}^{e}{\left(\frac{1}{\left|P_{h}\right|}\sum_{i\in P_{h}}{\left(\omega_{i}a_{i}\right)}^{p}\left(\frac{1}{|P_{h}|-1}\sum_{\begin{array}{c} j\in P_{h}\\ j\neq i \end{array}}{\left(\omega_{j}a_{j}\right)}^{q}\right)\right)}^{\frac{1}{p+q}}\right),\;\frac{1}{e}\left(\sum_{h=1}^{e}{\left(\frac{1}{\left|P_{h}\right|}\sum_{i\in P_{h}}{\left(\omega_{i}b_{i}\right)}^{p}\left(\frac{1}{|P_{h}|-1}\sum_{\begin{array}{c} j\in P_{h}\\ j\neq i \end{array}}{\left(\omega_{j}b_{j}\right)}^{q}\right)\right)}^{\frac{1}{p+q}}\right),\;\frac{1}{e}\left(\sum_{h=1}^{e}{\left(\frac{1}{\left|P_{h}\right|}\sum_{i\in P_{h}}{\left(\omega_{i}c_{i}\right)}^{p}\left(\frac{1}{|P_{h}|-1}\sum_{\begin{array}{c} j\in P_{h}\\ j\neq i \end{array}}{\left(\omega_{j}c_{j}\right)}^{q}\right)\right)}^{\frac{1}{p+q}}\right),\;\frac{1}{e}\left(\sum_{h=1}^{e}{\left(\frac{1}{\left|P_{h}\right|}\sum_{i\in P_{h}}{\left(\omega_{i}d_{i}\right)}^{p}\left(\frac{1}{|P_{h}|-1}\sum_{\begin{array}{c} j\in P_{h}\\ j\neq i \end{array}}{\left(\omega_{j}d_{j}\right)}^{q}\right)\right)}^{\frac{1}{p+q}}\right)],s_{\underset{\begin{array}{c} i,j\\ i\neq j \end{array}}{\min}(\theta_{i},\theta_{j})})$$

The proof of this theorem is similar with Theorem 4, it is omitted here.

**Theorem** **9.** *(Commutativity) Let* $\left({{\hat{s}}^{\prime }}_{1},{{\hat{s}}^{\prime }}_{2},\cdots ,{{\hat{s}}^{\prime }}_{n}\right)$ *be any permutation of* $\left({\hat{s}}_{1},{\hat{s}}_{2},\cdots ,{\hat{s}}_{n}\right)$*, then*

(30) $$\mathrm{TF}2\mathrm{DLWPBM}({{\hat{s}}^{\prime }}_{1},{{\hat{s}}^{\prime }}_{2},\cdots ,{{\hat{s}}^{\prime }}_{n})=\mathrm{TF}2\mathrm{DLWPBM}({\hat{s}}_{1},{\hat{s}}_{2},\cdots ,{\hat{s}}_{n})$$

The proof of this theorem is similar with Theorem 5, it is omitted here.

**Theorem** **10.** *(Boundedness) The TF2DLWPBM operator lies between the max and min operators:*

(31) $$\min({\hat{s}}_{1},{\hat{s}}_{2},\cdots ,{\hat{s}}_{n})\leq \mathrm{TF}2\mathrm{DLWPBM}({\hat{s}}_{1},{\hat{s}}_{2},\cdots ,{\hat{s}}_{n})\leq \max({\hat{s}}_{1},{\hat{s}}_{2},\cdots ,{\hat{s}}_{n})$$

The proof of this theorem is similar with Theorem 7, it is omitted here.

But TF2DLWPBM aggregate operator has not the idempotency property. Then, we discuss some special cases of TF2DLWPBM aggregate operator as follows.

1. When *q* = 0
  (32) $${\mathrm{TF}2\mathrm{DLWPBM}}^{p,0}({\hat{s}}_{1},{\hat{s}}_{2},\cdots ,{\hat{s}}_{n})=\frac{1}{e}\left(\sum_{h=1}^{e}{\left(\frac{1}{\left|P_{h}\right|}\sum_{i\in P_{h}}{\left(\omega_{i}{\hat{s}}_{i}\right)}^{p}\right)}^{\frac{1}{p}}\right)\;=([\frac{1}{e}\left(\sum_{h=1}^{e}{\left(\frac{1}{\left|P_{h}\right|}\sum_{i\in P_{h}}{\left(\omega_{i}a_{i}\right)}^{p}\right)}^{\frac{1}{p}}\right),\frac{1}{e}\left(\sum_{h=1}^{e}{\left(\frac{1}{\left|P_{h}\right|}\sum_{i\in P_{h}}{\left(\omega_{i}b_{i}\right)}^{p}\right)}^{\frac{1}{p}}\right),\frac{1}{e}\left(\sum_{h=1}^{e}{\left(\frac{1}{\left|P_{h}\right|}\sum_{i\in P_{h}}{\left(\omega_{i}c_{i}\right)}^{p}\right)}^{\frac{1}{p}}\right),\frac{1}{e}\left(\sum_{h=1}^{e}{\left(\frac{1}{\left|P_{h}\right|}\sum_{i\in P_{h}}{\left(\omega_{i}d_{i}\right)}^{p}\right)}^{\frac{1}{p}}\right)],s_{\underset{\begin{array}{c} i \end{array}}{\min}\theta_{i}})$$
2. When *p* = 1, *q* = 0
  (33) $${\mathrm{TF}2\mathrm{DLWPBM}}^{1,0}({\hat{s}}_{1},{\hat{s}}_{2},\cdots ,{\hat{s}}_{n})=\frac{1}{e}\left(\sum_{h=1}^{e}\left(\frac{1}{\left|P_{h}\right|}\sum_{i\in P_{h}}\omega_{i}{\hat{s}}_{i}^{}\right)\right)\;=([\frac{1}{e}\sum_{h=1}^{e}\left(\frac{1}{\left|P_{h}\right|}\sum_{i\in P_{h}}\omega_{i}a_{i}^{}\right),\;\frac{1}{e}\sum_{h=1}^{e}\left(\frac{1}{\left|P_{h}\right|}\sum_{i\in P_{h}}\omega_{i}b_{i}^{}\right),\;\frac{1}{e}\sum_{h=1}^{e}\left(\frac{1}{\left|P_{h}\right|}\sum_{i\in P_{h}}\omega_{i}c_{i}^{}\right),\;\frac{1}{e}\sum_{h=1}^{e}\left(\frac{1}{\left|P_{h}\right|}\sum_{i\in P_{h}}\omega_{i}d_{i}^{}\right)],s_{\underset{\begin{array}{c} i \end{array}}{\min}\theta_{i}})$$
3. When *p* = 1, *q* = 1
  (34) $${\mathrm{TF}2\mathrm{DLWPBM}}^{1,1}({\hat{s}}_{1},{\hat{s}}_{2},\cdots ,{\hat{s}}_{n})=\frac{1}{e}\left(\sum_{h=1}^{e}{\left(\frac{1}{\left|P_{h}\right|}\sum_{i\in P_{h}}\omega_{i}{\hat{s}}_{i}^{}\left(\frac{1}{|P_{h}|-1}\sum_{\begin{array}{c} j\in P_{h}\\ j\neq i \end{array}}\omega_{j}{\hat{s}}_{j}^{}\right)\right)}^{\frac{1}{2}}\right)=([\frac{1}{e}\left(\sum_{h=1}^{e}{\left(\frac{1}{\left|P_{h}\right|}\sum_{i\in P_{h}}\omega_{i}a_{i}^{}\left(\frac{1}{|P_{h}|-1}\sum_{\begin{array}{c} j\in P_{h}\\ j\neq i \end{array}}\omega_{j}a_{j}^{}\right)\right)}^{\frac{1}{2}}\right),\frac{1}{e}\left(\sum_{h=1}^{e}{\left(\frac{1}{\left|P_{h}\right|}\sum_{i\in P_{h}}\omega_{i}b_{i}^{}\left(\frac{1}{|P_{h}|-1}\sum_{\begin{array}{c} j\in P_{h}\\ j\neq i \end{array}}\omega_{j}b_{j}^{}\right)\right)}^{\frac{1}{2}}\right),\frac{1}{e}\left(\sum_{h=1}^{e}{\left(\frac{1}{\left|P_{h}\right|}\sum_{i\in P_{h}}\omega_{i}c_{i}^{}\left(\frac{1}{|P_{h}|-1}\sum_{\begin{array}{c} j\in P_{h}\\ j\neq i \end{array}}\omega_{j}c_{j}^{}\right)\right)}^{\frac{1}{2}}\right),\frac{1}{e}\left(\sum_{h=1}^{e}{\left(\frac{1}{\left|P_{h}\right|}\sum_{i\in P_{h}}\omega_{i}d_{i}^{}\left(\frac{1}{|P_{h}|-1}\sum_{\begin{array}{c} j\in P_{h}\\ j\neq i \end{array}}\omega_{j}d_{j}^{}\right)\right)}^{\frac{1}{2}}\right)],s_{\underset{\begin{array}{c} i,j\\ i\neq j \end{array}}{\min}(\theta_{i},\theta_{j})})$$

## 4. A Multiple Attribute Group Decision-Making Method Based on TF2DLWPBM Operator

Consider a MAGDM problem in the context of trapezoidal fuzzy two-dimension linguistic information: Suppose that there is a group of decision makers $\left\{D_{1},D_{2},\ldots D_{p}\right\}$, and $X=\left\{X_{1},X_{2},\ldots X_{m}\right\}$ be a set of *m* alternatives, $G=\left\{G_{1},G_{2},\ldots G_{n}\right\}$ be a set of attributes aims to choose the best alternative among m alternatives. The *n* attributes are partitioned into e class $P=\left\{P_{1},P_{2},\ldots P_{e}\right\}$, attribute only have interrelationship with rest attributes in the same partition. $\omega_{j}$ is the weight of the attributes from each partition, $\omega_{j}\geq 0$, $\sum_{j=1}^{n}\omega_{j}=1$. $\gamma_{k}\left(k=1,2,\ldots ,p\right)$ is a weight of decision makers $D_{\gamma }$, $\gamma_{k}\geq 0$, $\sum_{k=1}^{p}\gamma_{k}=1$.

Suppose that ${\hat{R}}^{k}={\left[{\hat{r}}_{ij}^{k}\right]}_{m\times n}$ is the decision matrix where ${\hat{r}}_{ij}^{k}=\left(\left[a_{ijk}.b_{ijk},c_{ijk},d_{ijk}\right],s_{\theta_{ij}^{k}}\right)$ represents the structure of the trapezoidal fuzzy two-dimension linguistic variable, and $a_{ijk}\leq b_{ijk}\leq c_{ijk}\leq d_{ijk}$,$\;s_{\theta_{ij}^{k}}\in S$, which shows that the decision maker $D_{k}$ gives an evaluation of the alternative $X_{i}$ through the analysis of the attribute $G_{j}$. Then, we can assemble the above information and rank the order of alternatives. 

In general, considering the directionality of attributes, ensuring benefit attribute and cost attribute have the same directionality. And different decision makers or different decision making methods may result in different evaluation criteria, so we need normalize class I information of trapezoidal fuzzy two-dimensional linguistic variables before using the TF2DLWPBM operator to aggregate the assessment information.

The steps of this method are as follows:

**Step 1.** Normalize the trapezoidal fuzzy two-dimensional linguistic variables.

Suppose ${\hat{V}}^{k}={\left[{\hat{v}}_{ij}^{k}\right]}_{m\times n}$ is the normalized matrix of decision matrix ${\hat{R}}^{k}={\left[{\hat{r}}_{ij}^{k}\right]}_{m\times n}$ where ${\hat{v}}_{ij}^{k}=(\left[a_{ij}^{k^{\prime }},b_{ij}^{k^{\prime }},c_{ij}^{k^{\prime }},d_{ij}^{k^{\prime }}\right],s_{\theta_{ij}^{k}})$, then the normalization method is chosen as follows:

1. For benefit attributes:

(35) $$[a_{ij}^{k}{}^{\prime },b_{ij}^{k}{}^{\prime },c_{ij}^{k}{}^{\prime },d_{ij}^{k}{}^{\prime }]=\left[\frac{a_{ij}^{k}}{Y},\frac{b_{ij}^{k}}{Y},\frac{c_{ij}^{k}}{Y},\frac{d_{ij}^{k}}{Y}\right]$$

where $Y=\sqrt{\sum_{i=1}^{m}\left[{\left(a_{ij}^{k}\right)}^{2}+{\left(b_{ij}^{k}\right)}^{2}+{\left(c_{ij}^{k}\right)}^{2}+{\left(d_{ij}^{k}\right)}^{2}\right]}$

2. For cost attributes:

(36) $$[a_{ij}^{k}{}^{\prime },b_{ij}^{k}{}^{\prime },c_{ij}^{k}{}^{\prime },d_{ij}^{k}{}^{\prime }]=\left[\frac{1/a_{ij}^{k}}{Z},\frac{1/b_{ij}^{k}}{Z},\frac{1/c_{ij}^{k}}{Z},\frac{1/d_{ij}^{k}}{Z}\right]$$

where $Z=\sqrt{\sum_{i=1}^{m}\left[{\left(1/a_{ij}^{k}\right)}^{2}+{\left(1/b_{ij}^{k}\right)}^{2}+{\left(1/c_{ij}^{k}\right)}^{2}+{\left(1/d_{ij}^{k}\right)}^{2}\right]}$

**Step 2.** Aggregate the assessment information of each decision maker by weighting method.

Suppose $U^{k}={\left[{\hat{u}}_{ij}\right]}_{m\times n}$ is the group decision matrix calculated from normalized matrix ${\hat{V}}^{k}={\left[{\hat{v}}_{ij}^{k}\right]}_{m\times n}$ by a weighting method, where ${\hat{u}}_{ij}=\left(\left[a_{ij}{}^{\prime \prime },b_{ij}{}^{\prime \prime },c_{ij}{}^{\prime \prime },d_{ij}{}^{\prime \prime }\right],s_{\theta_{ij}}\right)$, as follows:

(37) $$[a_{ij}^{}{}^{\prime \prime },b_{ij}^{}{}^{\prime \prime },c_{ij}^{}{}^{\prime \prime },d_{ij}^{}{}^{\prime \prime }]=\left[\sum_{k=1}^{p}\gamma_{k}a_{ij}^{k}{}^{\prime },\sum_{k=1}^{p}\gamma_{k}b_{ij}^{k}{}^{\prime },\sum_{k=1}^{p}\gamma_{k}c_{ij}^{k}{}^{\prime },\sum_{k=1}^{p}\gamma_{k}d_{ij}^{k}{}^{\prime }\right]$$

**Step 3.** Calculate the comprehensive evaluation value of each alternative.

We have obtained weights of the attributes, and the weights are expressed in exact numerical values. Then, we calculate the comprehensive group overall opinions by TF2DLWPBM operator, where ${\hat{u}}_{i}=\left(\left[a_{i},b_{i},c_{i},d_{i}\right],s_{\theta_{i}}\right)$ as follows:

(38) $$[a_{i}^{},b_{i}^{},c_{i}^{},d_{i}^{}]=\mathrm{TF}2\mathrm{DLWPBM}({\hat{u}}_{i1},{\hat{u}}_{i2},\cdots ,{\hat{u}}_{in})=([\frac{1}{e}\left(\sum_{h=1}^{e}{\left(\frac{1}{\left|P_{h}\right|}\sum_{j_{1}\in P_{h}}{\left(\omega_{j_{1}}a_{ij_{1}}^{}{}^{\prime \prime }\right)}^{p}\left(\frac{1}{|P_{h}|-1}\sum_{\begin{array}{c} j_{2}\in P_{h}\\ j_{2}\neq j_{1} \end{array}}{\left(\omega_{j_{2}}a_{ij_{2}}^{}{}^{\prime \prime }\right)}^{q}\right)\right)}^{\frac{1}{p+q}}\right),\frac{1}{e}\left(\sum_{h=1}^{e}{\left(\frac{1}{\left|P_{h}\right|}\sum_{j_{1}\in P_{h}}{\left(\omega_{j_{1}}b_{ij_{1}}^{}{}^{\prime \prime }\right)}^{p}\left(\frac{1}{|P_{h}|-1}\sum_{\begin{array}{c} j_{2}\in P_{h}\\ j_{2}\neq j_{1} \end{array}}{\left(\omega_{j_{2}}b_{ij_{2}}^{}{}^{\prime \prime }\right)}^{q}\right)\right)}^{\frac{1}{p+q}}\right),\frac{1}{e}\left(\sum_{h=1}^{e}{\left(\frac{1}{\left|P_{h}\right|}\sum_{j_{1}\in P_{h}}{\left(\omega_{j_{1}}c_{ij_{1}}^{}{}^{\prime \prime }\right)}^{p}\left(\frac{1}{|P_{h}|-1}\sum_{\begin{array}{c} j_{2}\in P_{h}\\ j_{2}\neq j_{1} \end{array}}{\left(\omega_{j_{2}}c_{ij_{2}}^{}{}^{\prime \prime }\right)}^{q}\right)\right)}^{\frac{1}{p+q}}\right),\frac{1}{e}\left(\sum_{h=1}^{e}{\left(\frac{1}{\left|P_{h}\right|}\sum_{j_{1}\in P_{h}}{\left(\omega_{j_{1}}d_{ij_{1}}^{}{}^{\prime \prime }\right)}^{p}\left(\frac{1}{|P_{h}|-1}\sum_{\begin{array}{c} j_{2}\in P_{h}\\ j_{2}\neq j_{1} \end{array}}{\left(\omega_{j_{2}}d_{ij_{2}}^{}{}^{\prime \prime }\right)}^{q}\right)\right)}^{\frac{1}{p+q}}\right)],s_{\underset{\begin{array}{c} j_{1},j_{2}\\ j_{1}\neq j_{2} \end{array}}{\min}(\theta_{j_{1}},\theta_{j_{2}})})$$

**Step 4.** Calculate the expectation of all alternatives’ TF2DLWPBM operators:

(39) $${\hat{f}}_{i}=E({\hat{u}}_{i})=\frac{a_{i}^{}+b_{i}^{}+c_{i}^{}+d_{i}^{}}{4}\cdot \frac{\theta_{i}}{l-1}$$

**Step 5.** Rank the alternatives.

Sort the alternatives by comparing the size of ${\hat{f}}_{i}\left(i=1,2,\ldots ,m\right)$. If the $\hat{f}$ of the alternative is greater than the $\hat{f}$ of the other alternatives, then the alternative is the best solution of all. On the contrary, the smallest $\hat{f}$ corresponding to the worst alternative of all alternatives.

## 5. An Illustrated Example

Many researchers and scholars in the fields of environmental health sciences or public health sciences have done a lot of work to study the damage to human beings caused by environmental pollution, analyzing the reasons and trying to find pollution reduction methods [43,44,45,46,47]. However, many of them have met with the difficulties of lack of data, like observations of ocean pollution or air quality, in time. The cost to obtain those data is not affordable because of technology restraints, not to mention the need for timely data. Under some circumstances where the pollution data for environment evaluation are not available, experts from different walks are needed to do the field research and help with the decision making process. It is quite helpful to apply the method based on the TF2DLWPBM operator to resolve the complex situation in such circumstances. Mathematics help the human thinking process be more systematic and reasonable, to make good decisions. Here we give an example of how the proposed method is applied to evaluation of river basin ecosystem health.

An environmental institute that needs to evaluate four river basins (*X*_1_,*X*_2_,*X*_3_,*X*_4_) and choose the healthiest ecosystem from among them to carry out further research. For further details of the four river basins, the environment institute’s management set up a team of three decision makers with weight vector *γ* =(0.4,0.35,0.25)*^T^*. The three decision makers should evaluate the comprehensive strength of the four river basins by considering the following five aspects:

*G*_1_: The unhealthy resources and consumption restrictions
*G*_2_: The sustainable development of the river basin’s economy
*G*_3_: People’s environmental awareness in the river basin
*G*_4_: The species diversity in the river basin
*G*_5_: The resilience of the river basin

In the evaluation process, because people’s thinking is subjective and complex, the expert evaluation results are fuzzy. Meanwhile, due to the fact the field of expert research is different and the investigated information is not complete, it is necessary for experts to evaluate the reliability of their evaluation results. Considering the above factors, we describe the expert information in the form of the trapezoidal fuzzy two-dimensional linguistic variable. The class I information is expressed by trapezoidal fuzzy number, and the class II information is expressed by a linguistic variable, *l* = 7, *S* = {*s*_0_,*s*_1_,*s*_2_,*s*_3_,*s*_4_,*s*_5_,*s*_6_) = {extremely unreliable, unreliable, fair, slightly reliable, reliable, extremely reliable}.

The attributes *G*_1_, *G*_2_ and *G*_3_ assess the health of the ecosystem in the river basins from the perspective of social development and human health. The attributes *G*_4_ and *G*_5_ measure the health of ecosystem in the river basins from the perspective of healthy development of ecological environment. We consider the interrelationship among the five attributes and partition the five attributes into two attribute sets. These attribute sets are *P*_1_ = {*G*_1_, *G*_1_, *G*_1_} and *P*_2_ = {*G*_4_, *G*_5_} with the weight vector *ω* = (0.2,0.15,0.15,0.3,0.2)*^T^*.

### 5.1. The Evaluation Steps

The evaluation steps for the four river basins are as follows:

The three decision makers give their decision matrixes as Table 1, Table 2 and Table 3.

Calculate the normalized matrixes based on Equations (35) and (36) as Table 4, Table 5 and Table 6.

Aggregate the assessment information for each alternative evaluated from each decision maker by weighting method. So we can get an aggregated matrix ${\hat{U}}^{k}$, which is shown in Table 7:

Calculate the comprehensive group overall opinions by TF2DLWPBM operator, suppose q=1, q=0 the results as follows:

$${\hat{u}}_{1}=\left(\left[0.0502,0.0809,0.1105,0.1499\right],s_{1}\right)$$

$${\hat{u}}_{2}=\left(\left[0.0401,0.0682,0.1047,0.1343\right],s_{1}\right)$$

$${\hat{u}}_{3}=\left(\left[0.0476,0.0746,0.1042,0.1391\right],s_{1}\right)$$

$${\hat{u}}_{4}=\left(\left[0.0408,0.0689,0.1027,0.1406\right],s_{2}\right)$$

Calculate expectations about each ${\hat{u}}_{i}\left(i=1,2,\ldots ,m\right)$, based on Equation (39).

For example, we can calculate the expectation ${\hat{f}}_{1}=E\left({\hat{u}}_{1}\right)$ and the calculative process is as follows:

$${\hat{f}}_{1}=E\left({\hat{u}}_{1}\right)=\frac{0.0502+0.0809+0.1105+0.1499}{4}\times \frac{1}{7-1}=0.0163$$

and the other expectations are below:

$$E\left({\hat{u}}_{2}\right)=0.0145,\;E\left({\hat{u}}_{3}\right)=0.0152,\;E\left({\hat{u}}_{4}\right)=0.0294$$

Determine the final ranking based on the comparison method described in SECT4, the result is

$$X_{4}≻X_{1}≻X_{3}≻X_{2}$$

### 5.2. Discussion

Parameters p and q can be used to indicate the degree of positive attitude of the decision makers, when decision makers are more optimistic in attributes variables, they can choose larger parameters to evaluate the alternatives. On the contrary, when decision makers are more pessimistic, they can choose the smaller parameters to obtain evaluation results.

To demonstrate the effect of the parameters p and q on evaluation results, we can analyze the rankings of the above problem as follows: in general, p and q can take any values in between 0 to ∞, we use some different special values in between 0 to 5, the ranking results are shown in Table 8.

From Table 8, the ordering of the alternatives is $X_{4}≻X_{1}≻X_{3}≻X_{2}$, there is no any change with the different special values of *p* or *q* between 0 to 4. However, when *p* = 1 and *q* = 5 or *q* = 1, *p* = 5, the order of $X_{2}$ and $X_{3}$ is reversed and the final ranking order is $X_{4}≻X_{1}≻X_{2}≻X_{3}$. Observe closely, the expectations of $X_{2}$ and $X_{3}$ are almost equal in size under the different parameters p and q. And small changes between them may be caused by different parameters. Then, we expand the value of p or q, and observe the changes about ranking order of the alternatives.

**Figure 1:**
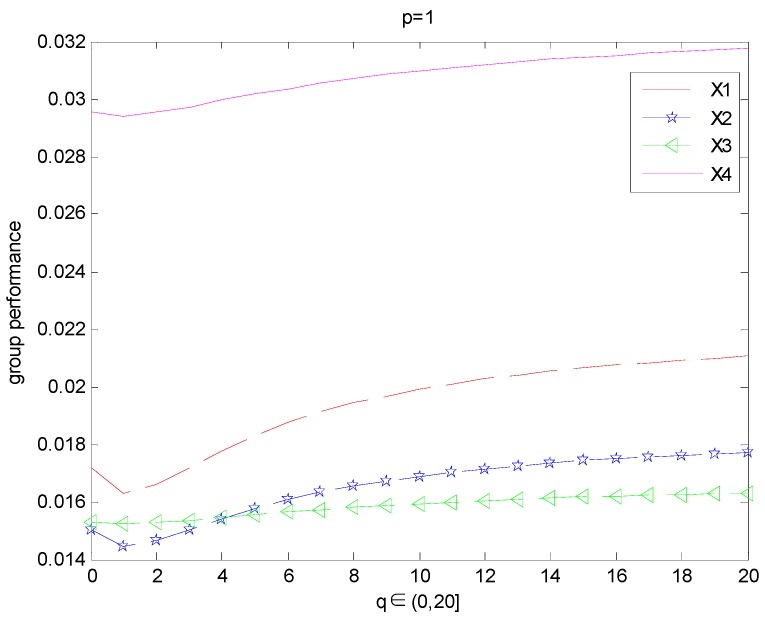
Group performance of the alternatives when p=1, and q∈(0,20].

**Figure 2:**
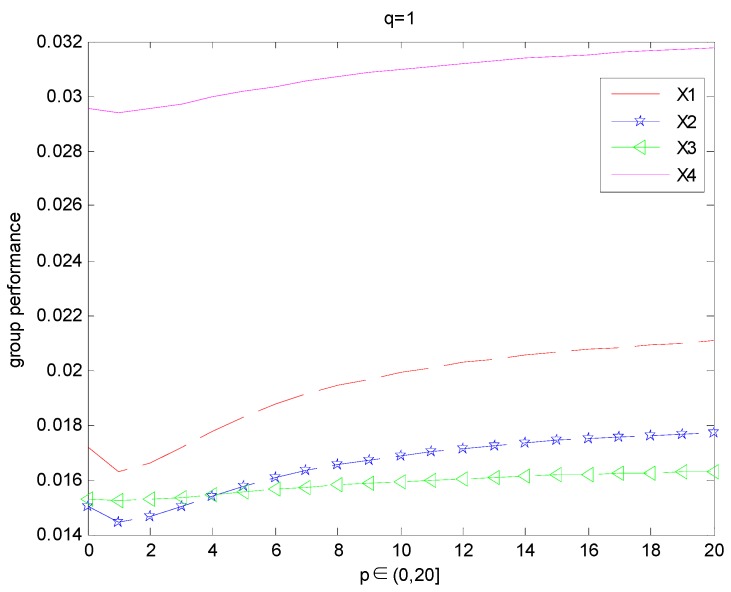
Group performance of the alternatives when q=1, and p∈(0,20].

For p=1 or q=1, if we take different values of q(p) experts’ overall opinions about the alternatives $X_{i}\left(i=1,2,3,4\right)$ are changed which is presented in Figure 1 (Figure 2).

From the abovementioned Figure 1 and Figure 2, we can observe that the results of ranking order depends on the value of the parameters *q* or *p* if we fix the value of the parameter *p* = 1 or *q* = 1:

1. When *p* = 1 and *q <* 4.1 or *q* = 1, *p <* 4.1, the ordering of alternatives is $X_{4}≻X_{1}≻X_{3}≻X_{2}$, the best alternative is $X_{4}$.
2. When *p* = 1 and *q* = 4.1 or *q* = 1, *p* = 4.1, the ordering of alternatives is $X_{4}≻X_{1}≻X_{3}=X_{2}$.
3. When *p* = 1 and *q >* 4.1 or *q* = 1, *p >* 4.1, the ordering of alternatives is $X_{4}≻X_{1}≻X_{2}≻X_{3}$, the best alternative is $X_{4}$.

**Figure 3:**
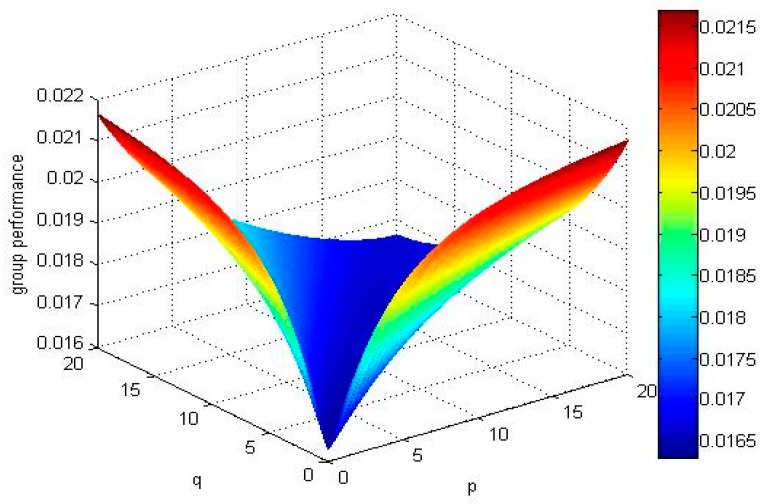
Group performance of when q,p∈(0, 20].

**Figure 4:**
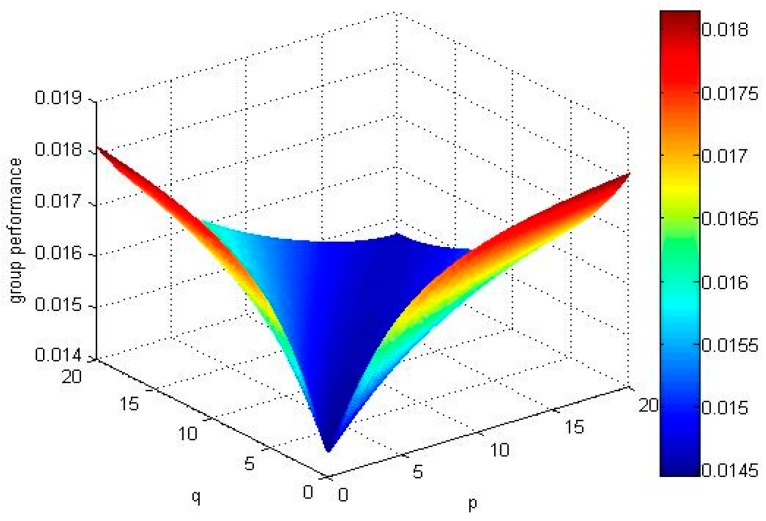
Group performance of $X_{2}$ when q,p∈(0, 20].

**Figure 5:**
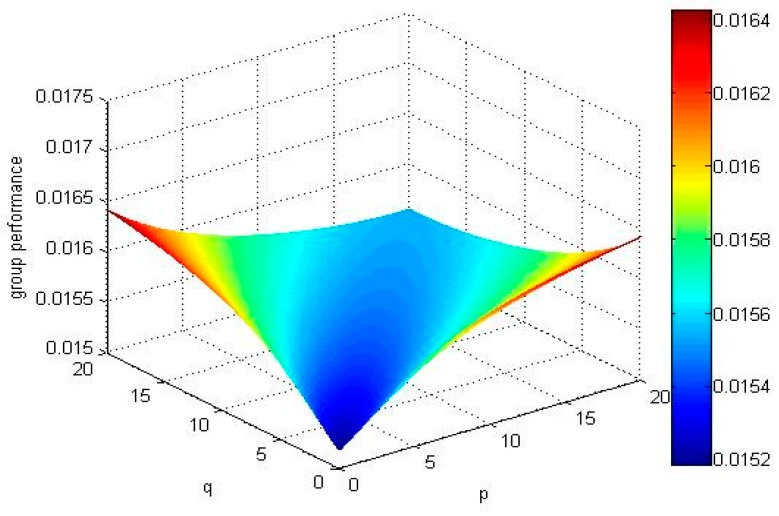
Group performance of $X_{3}$ when q,p∈(0, 20].

**Figure 6:**
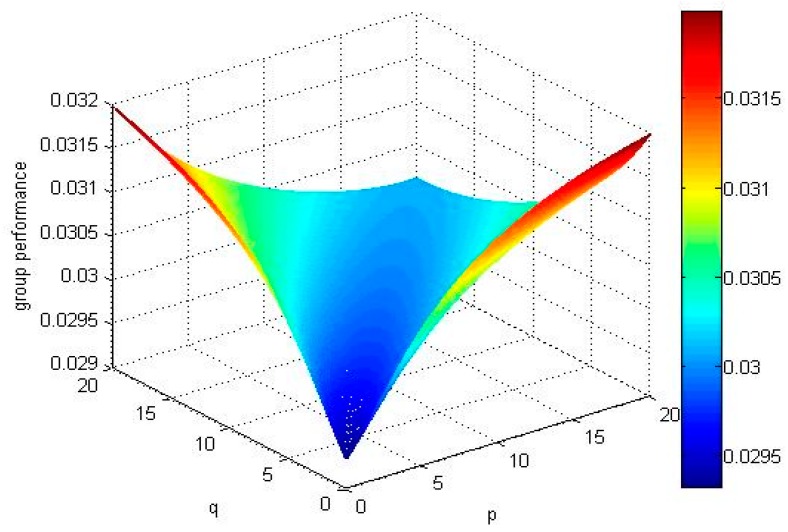
Group performance of $X_{4}$ when q,p∈(0, 20].

If we select different values of parameters, and calculate the expectation of all alternatives’ TF2DLWPBM operators and draw group performance of the alternatives in Figure 3, Figure 4, Figure 5 and Figure 6.

No matter how parameters *p* and *q* change, the alternative $X_{4}$ and alternative $X_{1}$ are the best two alternatives by comparing the above mentioned Figure 3, Figure 4, Figure 5 and Figure 6. The ranking order of the alternative $X_{2}$ and alternative $X_{3}$ changes with the absolute value of the difference between parameters *p* and *q*. If the absolute value of the difference between parameters *p* and *q* is small, , the ordering of alternatives is $X_{4}≻X_{1}≻X_{3}≻X_{2}$. If the absolute value of the difference between parameters *p* and *q* is big, $E\left({\hat{u}}_{2}\right)>E\left({\hat{u}}_{3}\right)$, the ordering of alternatives is $X_{4}≻X_{1}≻X_{2}≻X_{3}$.

### 5.3. Comparison with the Other Methods

To illustrate the effectiveness and superiority of the proposed method, we finish our comparison by utilizing the four methods. Table 9 shows the ranking orders of the alternatives obtained by the four existing methods are significantly different from the ranking order obtained by the proposed method.

To verify the effectiveness of the proposed decision making method, we made use of the example that an environment institute choose the healthiest ecosystem compare the method introduced by Dutta [29], we translated the trapezoidal fuzzy numbers in class I into linguistic variables by the approach proposed by Liu [48], and calculate results and rank alternatives by using the method proposed by Dutta. The ranking: $X_{4}≻X_{1}≻X_{3}≻X_{2}$ is the same result compared with calculating by TF2DLWPBM operator. Second, we compared the method in this paper with method based on the TTFLWBM aggregation operator that combined trapezoidal fuzzy 2-dimensional linguistic information with BM operator introduced by Shi [49]. Calculating the expectation of all alternatives’ TTFLWBM and the final ranking is $X_{4}≻X_{1}≻X_{3}≻X_{2}$ when *p* = 1 and *q* = 1. In addition, comparing with the existing aggregation operators, results of the TF2DLPGA and the TF2DLPGWA operators proposed by Li [21] are same and the order of ranking is $X_{4}≻X_{1}≻X_{2}≻X_{3}$ when λ=2. The expectation of $X_{2}$ and $X_{3}$ are almost equal in size, this is consistent with the proposed method in this paper.

To demonstrate the superiority of this method, we compared the method based on the TTFLWBM aggregation operator developed by Shi [49]. For *p* = 1 or *q* = 1, we found the point that the expectations of the alternative $X_{2}$ and alternative $X_{3}$ are equal. When *p* = 1 and *q* = 2.9 or *q* = 1, *p* = 2.9, the rank ordering is $X_{4}≻X_{1}≻X_{3}=X_{2}$. In this paper, when *p* = 1 and *q* = 4.1 or *q* = 1, *p* = 4.1, the result is $X_{4}≻X_{1}≻X_{3}=X_{2}$. Obviously the method proposed by Shi is more sensitive, which is because Shi’s method did not take into the inter-relationship among the attributes. In the case of a small gap between the attributes, Shi’s method may be biased against the final results. In addition, the method introduced by Liu [18] was based on similarity measures, but it does not take the decision makers’ preferences in the model. The method in this paper has the capability to express people’s preferences by adjusting parameters *p* and *q* based on the aggregation operators, which increases the application range of method and improves the accuracy of the decision making problems.

## 6. Conclusions

In this study, we have developed a new method for solving MADGM in a fuzzy 2-dimensional linguistic environment. This research makes two main contributions. In the first phase, we applied the PBM in the fuzzy 2-dimensional linguistic environment, and then, we proposed the TF2DLPBM and TF2DLWPBM operators. The proposed operators can build the interrelationships among each attribute and have the capability to capture inter-relationships among the attributes after some preparatory work where attributes are partitioned into several unrelated classes and each attribute only has interrelationships with rest of the attributes in the same class. In the second phase, we developed a new method on the basis of the TF2DLWPBM operator to solve MAGDM problems. We applied the proposed method to a group decision making of selecting the healthiest ecosystem from among four river basins, and then, we discussed how to express the preferences of decision makers by adjusting the parameters in the operator and analyzed the relationship of different parameters with the final ranking of alternatives. The proposed method has also been compared with other existing methods to illustrate the effectiveness and superiority. 

It is worth emphasizing that the proposed method has following advantages: (1) describing the decision makers’ evolution by using the trapezoidal fuzzy two-dimensional linguistic variable which can improve accuracy and application range of decision makers’ descriptions of alternatives; (2) considering partitioned structure and calculating the inter-relationship among the attributes by TF2DLWPBM, the developed method is more scientific to do decision making; (3) adjusting the parameters on the basis of preferences of decision makers, the developed method is more flexible to complex environment.

The proposed method based on the TF2DLPBM and TF2DLWPBM operators is suitable to handle real-life problems where uncertainty is involved in the decision process. In the cases where interrelationships among each attribute are clear, the method always can calculate the interrelationships clearly and easily. However, in a complex system there may be inherent relationships among each attribute. In future, we will investigate the use of TF2DLPBM for solving the decision-making problem in complex interrelationship environments and illustrate some meaningful applications in the environmental health sciences and public health fields.

## Figures and Tables

**Table 1:** Decision matrix ${\hat{R}}^{1}$ .

	$G_{1}$	$G_{2}$	$G_{3}$	$G_{4}$	$G_{5}$
$X_{1}$	$\left(\left[2,3,5,6\right],s_{5}\right)$	$\left(\left[4,5,7,8\right],s_{2}\right)$	$\left(\left[2,3,5,6\right],s_{6}\right)$	$\left(\left[5,6,7,8\right],s_{4}\right)$	$\left(\left[1,2,3,4\right],s_{3}\right)$
$X_{2}$	$\left(\left[3,4,6,7\right],s_{3}\right)$	$\left(\left[2,4,6,7\right],s_{3}\right)$	$\left(\left[1,3,6,8\right],s_{1}\right)$	$\left(\left[3,5,6,7\right],s_{5}\right)$	$\left(\left[2,3,5,6\right],s_{4}\right)$
$X_{3}$	$\left(\left[2,4,5,8\right],s_{4}\right)$	$\left(\left[2,3,5,6\right],s_{5}\right)$	$\left(\left[2,4,5,6\right],s_{3}\right)$	$\left(\left[1,2,3,4\right],s_{1}\right)$	$\left(\left[3,5,6,7\right],s_{2}\right)$
$X_{4}$	$\left(\left[1,3,4,5\right],s_{6}\right)$	$\left(\left[3,5,6,7\right],s_{6}\right)$	$\left(\left[2,3,4,5\right],s_{5}\right)$	$\left(\left[1,3,4,5\right],s_{2}\right)$	$\left(\left[2,3,6,8\right],s_{2}\right)$

**Table 2:** Decision matrix ${\hat{R}}^{2}.$

	$G_{1}$	$G_{2}$	$G_{3}$	$G_{4}$	$G_{5}$
$X_{1}$	$\left(\left[1,2,4,5\right],s_{4}\right)$	$\left(\left[2,4,5,6\right],s_{3}\right)$	$\left(\left[3,5,6,8\right],s_{5}\right)$	$\left(\left[2,4,6,7\right],s_{6}\right)$	$\left(\left[1,3,4,5\right],s_{1}\right)$
$X_{2}$	$\left(\left[2,3,5,8\right],s_{5}\right)$	$\left(\left[3,5,6,7\right],s_{2}\right)$	$\left(\left[2,3,7,8\right],s_{1}\right)$	$\left(\left[1,3,5,8\right],s_{5}\right)$	$\left(\left[1,2,3,4\right],s_{4}\right)$
$X_{3}$	$\left(\left[2,3,5,6\right],s_{1}\right)$	$\left(\left[2,3,5,7\right],s_{4}\right)$	$\left(\left[2,4,5,7\right],s_{2}\right)$	$\left(\left[3,4,5,6\right],s_{3}\right)$	$\left(\left[2,3,5,6\right],s_{5}\right)$
$X_{4}$	$\left(\left[4,5,7,8\right],s_{3}\right)$	$\left(\left[4,6,7,8\right],s_{5}\right)$	$\left(\left[3,4,6,7\right],s_{4}\right)$	$\left(\left[2,3,4,5\right],s_{2}\right)$	$\left(\left[3,5,6,8\right],s_{2}\right)$

**Table 3:** Decision matrix ${\hat{R}}^{3}.$

	$G_{1}$	$G_{2}$	$G_{3}$	$G_{4}$	$G_{5}$
$X_{1}$	$\left(\left[2,4,5,7\right],s_{3}\right)$	$\left(\left[3,4,5,7\right],s_{1}\right)$	$\left(\left[2,3,5,7\right],s_{4}\right)$	$\left(\left[3,4,5,8\right],s_{6}\right)$	$\left(\left[3,5,6,8\right],s_{4}\right)$
$X_{2}$	$\left(\left[3,5,6,8\right],s_{1}\right)$	$\left(\left[2,4,6,7\right],s_{3}\right)$	$\left(\left[2,3,4,6\right],s_{5}\right)$	$\left(\left[2,3,6,7\right],s_{5}\right)$	$\left(\left[2,3,4,5\right],s_{3}\right)$
$X_{3}$	$\left(\left[1,3,5,6\right],s_{2}\right)$	$\left(\left[2,4,5,6\right],s_{4}\right)$	$\left(\left[3,4,6,8\right],s_{6}\right)$	$\left(\left[2,3,5,6\right],s_{1}\right)$	$\left(\left[3,5,6,7\right],s_{6}\right)$
$X_{4}$	$\left(\left[2,5,6,7\right],s_{4}\right)$	$\left(\left[1,2,4,5\right],s_{2}\right)$	$\left(\left[1,2,4,5\right],s_{3}\right)$	$\left(\left[1,2,5,6\right],s_{3}\right)$	$\left(\left[1,3,5,6\right],s_{2}\right)$

**Table 4:** Normalized decision matrix ${\hat{V}}^{1}.$

	$G_{1}$	$G_{2}$	$G_{3}$	$G_{4}$	$G_{5}$
$X_{1}$	$\left(\left[\begin{array}{c} 0.112,0.134,\\ 0.223,0.335 \end{array}\right],s_{5}\right)$	$\left(\left[\begin{array}{c} 0.118,0.235,\\ 0.329,0.376 \end{array}\right],s_{2}\right)$	$\left(\left[\begin{array}{c} 0.055,0.165,\\ 0.276,0.386 \end{array}\right],s_{6}\right)$	$\left(\left[\begin{array}{c} 0.259,0.310,\\ 0.362,0.414 \end{array}\right],s_{4}\right)$	$\left(\left[\begin{array}{c} 0.055,0.109,\\ 0.164,0.218 \end{array}\right],s_{4}\right)$
$X_{2}$	$\left(\left[\begin{array}{c} 0.096,0.112,\\ 0.167,0.223 \end{array}\right],s_{3}\right)$	$\left(\left[\begin{array}{c} 0.094,0.188,\\ 0.282,0.329 \end{array}\right],s_{3}\right)$	$\left(\left[\begin{array}{c} 0.055,0.165,\\ 0.331,0.441 \end{array}\right],s_{1}\right)$	$\left(\left[\begin{array}{c} 0.155,0.259,\\ 0.310,0.362 \end{array}\right],s_{5}\right)$	$\left(\left[\begin{array}{c} 0.109,0.164,\\ 0.273,0.327 \end{array}\right],s_{5}\right)$
$X_{3}$	$\left(\left[\begin{array}{c} 0.084,0.134\\ 0.167,0.335 \end{array}\right],s_{4}\right)$	$\left(\left[\begin{array}{c} 0.094,0.141,\\ 0.235,0.329 \end{array}\right],s_{5}\right)$	$\left(\left[\begin{array}{c} 0.110,0.221,\\ 0.276,0.331 \end{array}\right],s_{3}\right)$	$\left(\left[\begin{array}{c} 0.052,0.103,\\ 0.155,0.207 \end{array}\right],s_{1}\right)$	$\left(\left[\begin{array}{c} 0.164,0.273,\\ 0.327,0.382 \end{array}\right],s_{1}\right)$
$X_{4}$	$\left(\left[\begin{array}{c} 0.114,0.167,\\ 0.223,0.669 \end{array}\right],s_{6}\right)$	$\left(\left[\begin{array}{c} 0.141,0.235\\ 0.282,0.329 \end{array}\right],s_{6}\right)$	$\left(\left[\begin{array}{c} 0.110,0.165,\\ 0.221,0.276 \end{array}\right],s_{5}\right)$	$\left(\left[\begin{array}{c} 0.052,0.155,\\ 0.207,0.259 \end{array}\right],s_{2}\right)$	$\left(\left[\begin{array}{c} 0.109,0.164,\\ 0.327,0.436 \end{array}\right],s_{2}\right)$

**Table 5:** Normalized decision matrix ${\hat{V}}^{2}.$

		$G_{2}$	$G_{3}$	$G_{4}$	$G_{5}$
$X_{1}$	$\left(\left[\begin{array}{c} 0.131,0.164,\\ 0.327,0.654 \end{array}\right],s_{4}\right)$	$\left(\left[\begin{array}{c} 0.094,0.188,\\ 0.235,0.282 \end{array}\right],s_{3}\right)$	$\left(\left[\begin{array}{c} 0.139,0.232,\\ 0.279,0.371 \end{array}\right],s_{5}\right)$	$\left(\left[\begin{array}{c} 0.107,0.216,\\ 0.323,0.377 \end{array}\right],s_{6}\right)$	$\left(\left[\begin{array}{c} 0.059,0.177,\\ 0.235,0.294 \end{array}\right],s_{1}\right)$
$X_{2}$	$\left(\left[\begin{array}{c} 0.082,0.131,\\ 0.218,0.327 \end{array}\right],s_{5}\right)$	$\left(\left[\begin{array}{c} 0.141,0.235,\\ 0.282,0.329 \end{array}\right],s_{2}\right)$	$\left(\left[\begin{array}{c} 0.093,0.139,\\ 0.325,0.371 \end{array}\right],s_{1}\right)$	$\left(\left[\begin{array}{c} 0.054,0.162,\\ 0.270,0.431 \end{array}\right],s_{5}\right)$	$\left(\left[\begin{array}{c} 0.059,0.118,\\ 0.177,0.235 \end{array}\right],s_{4}\right)$
$X_{3}$	$\left(\left[\begin{array}{c} 0.109,0.131,\\ 0.218,0.327 \end{array}\right],s_{1}\right)$	$\left(\left[\begin{array}{c} 0.094,0.141,\\ 0.235,0.329 \end{array}\right],s_{4}\right)$	$\left(\left[\begin{array}{c} 0.093,0.186,\\ 0.232,0.325 \end{array}\right],s_{2}\right)$	$\left(\left[\begin{array}{c} 0.162,0.216,\\ 0.270,0.324 \end{array}\right],s_{3}\right)$	$\left(\left[\begin{array}{c} 0.118,0.177,\\ 0.294,0.353 \end{array}\right],s_{5}\right)$
$X_{4}$	$\left(\left[\begin{array}{c} 0.082,0.094,\\ 0.131,0.164 \end{array}\right],s_{3}\right)$	$\left(\left[\begin{array}{c} 0.141,0.235,\\ 0.329,0.376 \end{array}\right],s_{5}\right)$	$\left(\left[\begin{array}{c} 0.139,0.186,\\ 0.279,0.325 \end{array}\right],s_{4}\right)$	$\left(\left[\begin{array}{c} 0.108,0.162,\\ 0.216,0.270 \end{array}\right],s_{2}\right)$	$\left(\left[\begin{array}{c} 0.177,0.294,\\ 0.353,0.471 \end{array}\right],s_{2}\right)$

**Table 6:** Normalized decision matrix ${\hat{V}}^{3}.$

	$G_{1}$	$G_{2}$	$G_{3}$		$G_{4}$	$G_{5}$
$X_{1}$	$\left(\left[\begin{array}{c} 0.099,0.139,\\ 0.173,0.346 \end{array}\right],s_{3}\right)$	$\left(\left[\begin{array}{c} 0.165,0.220,\\ 0.275,0.385 \end{array}\right],s_{1}\right)$	$\left(\left[\begin{array}{c} 0.111,0.167,\\ 0.278,0.389 \end{array}\right],s_{4}\right)$		$\left(\left[\begin{array}{c} 0.160,0.213,\\ 0.267,0.426 \end{array}\right],s_{6}\right)$	$\left(\left[\begin{array}{c} 0.154,0.257,\\ 0.309,0.412 \end{array}\right],s_{4}\right)$
$X_{2}$	$\left(\left[\begin{array}{c} 0.087,0.115,\\ 0.139,0.231 \end{array}\right],s_{1}\right)$	$\left(\left[\begin{array}{c} 0.110,0.220,\\ 0.330,0.385 \end{array}\right],s_{3}\right)$	$\left(\left[\begin{array}{c} 0.111,0.167,\\ 0.223,0.334 \end{array}\right],s_{5}\right)$		$\left(\left[\begin{array}{c} 0.107,0.160,\\ 0.320,0.373 \end{array}\right],s_{5}\right)$	$\left(\left[\begin{array}{c} 0.103,0.154,\\ 0.177,0.257 \end{array}\right],s_{3}\right)$
$X_{3}$	$\left(\left[\begin{array}{c} 0.115,0.139,\\ 0.231,0.693 \end{array}\right],s_{2}\right)$	$\left(\left[\begin{array}{c} 0.110,0.220,\\ 0.275,0.330 \end{array}\right],s_{4}\right)$	$\left(\left[\begin{array}{c} 0.167,0.223,\\ 0.334,0.445 \end{array}\right],s_{6}\right)$		$\left(\left[\begin{array}{c} 0.106,0.160,\\ 0.267,0.312 \end{array}\right],s_{1}\right)$	$\left(\left[\begin{array}{c} 0.154,0.257,\\ 0.309,0.360 \end{array}\right],s_{6}\right)$
$X_{4}$	$\left(\left[\begin{array}{c} 0.099,0.115,\\ 0.139,0.346 \end{array}\right],s_{4}\right)$	$\left(\left[\begin{array}{c} 0.055,0.110,\\ 0.220,0.275 \end{array}\right],s_{2}\right)$	$\left(\left[\begin{array}{c} 0.056,0.111,\\ 0.222,0.278 \end{array}\right],s_{3}\right)$		$\left(\left[\begin{array}{c} 0.053,0.107,\\ 0.267,0.320 \end{array}\right],s_{3}\right)$	$\left(\left[\begin{array}{c} 0.051,0.154,\\ 0.257,0.309 \end{array}\right],s_{2}\right)$

**Table 7:** Aggregated matrix ${\hat{U}}^{k}.$

	$G_{1}$	$G_{2}$	$G_{3}$	$G_{4}$	$G_{5}$
$X_{1}$	$\left(\left[\begin{array}{c} 0.114,0.144,\\ 0.242,0.441 \end{array}\right],s_{3}\right)$	$\left(\left[\begin{array}{c} 0.152,0.215,\\ 0.284,0.349 \end{array}\right],s_{1}\right)$	$\left(\left[\begin{array}{c} 0.098,0.187,\\ 0.277,0.382 \end{array}\right],s_{4}\right)$	$\left(\left[\begin{array}{c} 0.183,0.253,\\ 0.323,0.406 \end{array}\right],s_{4}\right)$	$\left(\left[\begin{array}{c} 0.084,0.172,\\ 0.227,0.297 \end{array}\right],s_{4}\right)$
$X_{2}$	$\left(\left[\begin{array}{c} 0.089,0.18,\\ 0.175,0.258 \end{array}\right],s_{1}\right)$	$\left(\left[\begin{array}{c} 0.114,0.212,\\ 0.296,0.345 \end{array}\right],s_{2}\right)$	$\left(\left[\begin{array}{c} 0.083,0.157,\\ 0.299,0.389 \end{array}\right],s_{1}\right)$	$\left(\left[\begin{array}{c} 0.109,0.200,\\ 0.300,0.387 \end{array}\right],s_{5}\right)$	$\left(\left[\begin{array}{c} 0.091,0.146,\\ 0.223,0.278 \end{array}\right],s_{3}\right)$
$X_{3}$	$\left(\left[\begin{array}{c} 0.101,0.134,\\ 0.201,0.432 \end{array}\right],s_{1}\right)$	$\left(\left[\begin{array}{c} 0.099,0.163,\\ 0.264,0.311 \end{array}\right],s_{4}\right)$	$\left(\left[\begin{array}{c} 0.121,0.210,\\ 0.278,0.361 \end{array}\right],s_{2}\right)$	$\left(\left[\begin{array}{c} 0.102,0.155,\\ 0.223,0.276 \end{array}\right],s_{1}\right)$	$\left(\left[\begin{array}{c} 0.146,0.238,\\ 0.311,0.366 \end{array}\right],s_{1}\right)$
$X_{4}$	$\left(\left[\begin{array}{c} 0.107,0.129,\\ 0.170,0.417 \end{array}\right],s_{3}\right)$	$\left(\left[\begin{array}{c} 0.132,0.215,\\ 0.280,0.329 \end{array}\right],s_{2}\right)$	$\left(\left[\begin{array}{c} 0.104,0.157,\\ 0.240,0.292 \end{array}\right],s_{3}\right)$	$\left(\left[\begin{array}{c} 0.070,0.144,\\ 0.226,0.279 \end{array}\right],s_{2}\right)$	$\left(\left[\begin{array}{c} 0.115,0.203,\\ 0.316,0.412 \end{array}\right],s_{2}\right)$

**Table 8:** The ranking result of p and q in between 0 to 5.

*p*, *q*	$\hat{u_{i}}$	$E\left(\hat{u_{i}}\right)$	Ranking
*p* = 1 *q* = 0	${\hat{u}}_{1}=\left(\left[0.0559,0.0849,0.1154,0.1564\right],s_{1}\right)$	$E\left({\hat{u}}_{1}\right)=0.0172$	$X_{4}≻X_{1}≻X_{3}≻X_{2}$
	${\hat{u}}_{2}=\left(\left[0.0412,0.0710,0.1087,0.1398\right],s_{1}\right)$	$E\left({\hat{u}}_{2}\right)=0.0150$	
	${\hat{u}}_{3}=\left(\left[0.0467,0.0746,0.1042,0.1404\right],s_{1}\right)$	$E\left({\hat{u}}_{3}\right)=0.0153$	
	${\hat{u}}_{4}=\left(\left[0.0409,0.069,0.1028,0.1419\right],s_{2}\right)$	$E\left({\hat{u}}_{4}\right)=0.0296$	
*p* = 1 *q* = 1	${\hat{u}}_{1}=\left(\left[0.0502,0.0809,0.1105,0.1499\right],s_{1}\right)$	$E\left({\hat{u}}_{1}\right)=0.0163$	$X_{4}≻X_{1}≻X_{3}≻X_{2}$
	${\hat{u}}_{2}=\left(\left[0.0401,0.0682,0.1047,0.1343\right],s_{1}\right)$	$E\left({\hat{u}}_{2}\right)=0.0145$	
	${\hat{u}}_{3}=\left(\left[0.0476,0.0746,0.1042,0.1391\right],s_{1}\right)$	$E\left({\hat{u}}_{3}\right)=0.0152$	
	${\hat{u}}_{4}=\left(\left[0.0408,0.0689,0.1027,0.1406\right],s_{2}\right)$	$E\left({\hat{u}}_{4}\right)=0.0294$	
*p* = 1 *q* = 2	${\hat{u}}_{1}=\left(\left[0.0521,0.0822,0.1121,0.1526\right],s_{1}\right)$	$E\left({\hat{u}}_{1}\right)=0.0166$	$X_{4}≻X_{1}≻X_{3}≻X_{2}$
	${\hat{u}}_{2}=\left(\left[0.0405,0.0692,0.1061,0.1361\right],s_{1}\right)$	$E\left({\hat{u}}_{2}\right)=0.0147$	
	${\hat{u}}_{3}=\left(\left[0.0476,0.0746,0.1042,0.1402\right],s_{1}\right)$	$E\left({\hat{u}}_{3}\right)=0.0153$	
	${\hat{u}}_{4}=\left(\left[0.0409,0.0690,0.1028,0.1417\right],s_{2}\right)$	$E\left({\hat{u}}_{4}\right)=0.0295$	
*p* = 1 *q* = 4	${\hat{u}}_{1}=\left(\left[0.0585,0.0873,0.1183,0.1625\right],s_{1}\right)$	$E\left({\hat{u}}_{1}\right)=0.0178$	$X_{4}≻X_{1}≻X_{3}≻X_{2}$
	${\hat{u}}_{2}=\left(\left[0.0422,0.0731,0.1114,0.1432\right],s_{1}\right)$	$E\left({\hat{u}}_{2}\right)=0.0154$	
	${\hat{u}}_{3}=\left(\left[0.0479,0.0749,0.1043,0.1440\right],s_{1}\right)$	$E\left({\hat{u}}_{3}\right)=0.0155$	
	${\hat{u}}_{4}=\left(\left[0.0412,0.0694,0.1031,0.1456\right],s_{2}\right)$	$E\left({\hat{u}}_{4}\right)=0.0300$	
*p* = 1 *q* = 5	${\hat{u}}_{1}=\left(\left[0.0610,0.0896,0.1212,0.1673\right],s_{1}\right)$	$E\left({\hat{u}}_{1}\right)=0.0183$	$X_{4}≻X_{1}≻X_{2}≻X_{3}$
	${\hat{u}}_{2}=\left(\left[0.0430,0.0749,0.140,0.1465\right],s_{1}\right)$	$E\left({\hat{u}}_{2}\right)=0.0158$	
	${\hat{u}}_{3}=\left(\left[0.0480,0.0750,0.1044,0.1460\right],s_{1}\right)$	$E\left({\hat{u}}_{3}\right)=0.0156$	
	${\hat{u}}_{4}=\left(\left[0.0413,0.0697,0.1033,0.1476\right],s_{2}\right)$	$E\left({\hat{u}}_{4}\right)=0.0302$	
*p* = 2 *q* = 2	${\hat{u}}_{1}=\left(\left[0.0504,0.0809,0.1106,0.1506\right],s_{1}\right)$	$E\left({\hat{u}}_{1}\right)=0.0164$	$X_{4}≻X_{1}≻X_{3}≻X_{2}$
	${\hat{u}}_{2}=\left(\left[0.0402,0.0683,0.1048,0.1343\right],s_{1}\right)$	$E\left({\hat{u}}_{2}\right)=0.0145$	
	${\hat{u}}_{3}=\left(\left[0.0476,0.0746,0.1042,0.1399\right],s_{1}\right)$	$E\left({\hat{u}}_{3}\right)=0.0153$	
	${\hat{u}}_{4}=\left(\left[0.0409,0.0690,0.1028,0.1415\right],s_{2}\right)$	$E\left({\hat{u}}_{4}\right)=0.0295$	
*p* = 0 *q* = 1	${\hat{u}}_{1}=\left(\left[0.0559,0.0849,0.1154,0.1564\right],s_{1}\right)$	$E\left({\hat{u}}_{1}\right)=0.0172$	$X_{4}≻X_{1}≻X_{3}≻X_{2}$
	${\hat{u}}_{2}=\left(\left[0.0412,0.0710,0.1087,0.1398\right],s_{1}\right)$	$E\left({\hat{u}}_{2}\right)=0.0150$	
	${\hat{u}}_{3}=\left(\left[0.0467,0.0746,0.1042,0.1404\right],s_{1}\right)$	$E\left({\hat{u}}_{3}\right)=0.0153$	
	${\hat{u}}_{4}=\left(\left[0.0409,0.069,0.1028,0.1419\right],s_{2}\right)$	$E\left({\hat{u}}_{4}\right)=0.0296$	
*p* = 2 *q* = 1	${\hat{u}}_{1}=\left(\left[0.0521,0.0822,0.1121,0.1526\right],s_{1}\right)$	$E\left({\hat{u}}_{1}\right)=0.0166$	$X_{4}≻X_{1}≻X_{3}≻X_{2}$
	${\hat{u}}_{2}=\left(\left[0.0405,0.0692,0.1061,0.1361\right],s_{1}\right)$	$E\left({\hat{u}}_{2}\right)=0.0147$	
		$E\left({\hat{u}}_{3}\right)=0.0153$	
	${\hat{u}}_{4}=\left(\left[0.0409,0.0690,0.1028,0.1417\right],s_{2}\right)$	$E\left({\hat{u}}_{4}\right)=0.0295$	
*p* = 4 *q* = 1	${\hat{u}}_{1}=\left(\left[0.0585,0.0873,0.1183,0.1625\right],s_{1}\right)$	$E\left({\hat{u}}_{1}\right)=0.0178$	$X_{4}≻X_{1}≻X_{3}≻X_{2}$
	${\hat{u}}_{2}=\left(\left[0.0422,0.0731,0.1114,0.1432\right],s_{1}\right)$	$E\left({\hat{u}}_{2}\right)=0.0154$	
	${\hat{u}}_{3}=\left(\left[0.0479,0.0749,0.1043,0.1440\right],s_{1}\right)$	$E\left({\hat{u}}_{3}\right)=0.0155$	
	${\hat{u}}_{4}=\left(\left[0.0412,0.0694,0.1031,0.1456\right],s_{2}\right)$	$E\left({\hat{u}}_{4}\right)=0.0300$	
*p* = 5 *q* = 1	${\hat{u}}_{1}=\left(\left[0.0610,0.0896,0.1212,0.1673\right],s_{1}\right)$	$E\left({\hat{u}}_{1}\right)=0.0183$	$X_{4}≻X_{1}≻X_{2}≻X_{3}$
	${\hat{u}}_{2}=\left(\left[0.0430,0.0749,0.140,0.1465\right],s_{1}\right)$	$E\left({\hat{u}}_{2}\right)=0.0158$	
	${\hat{u}}_{3}=\left(\left[0.0480,0.0750,0.1044,0.1460\right],s_{1}\right)$	$E\left({\hat{u}}_{3}\right)=0.0156$	
	${\hat{u}}_{4}=\left(\left[0.0413,0.0697,0.1033,0.1476\right],s_{2}\right)$	$E\left({\hat{u}}_{4}\right)=0.0302$	
*p* = 2 *q* = 2	${\hat{u}}_{1}=\left(\left[0.0504,0.0809,0.1106,0.1506\right],s_{1}\right)$	$E\left({\hat{u}}_{1}\right)=0.0164$	$X_{4}≻X_{1}≻X_{3}≻X_{2}$
	${\hat{u}}_{2}=\left(\left[0.0402,0.0683,0.1048,0.1343\right],s_{1}\right)$	$E\left({\hat{u}}_{2}\right)=0.0145$	
	${\hat{u}}_{3}=\left(\left[0.0476,0.0746,0.1042,0.1399\right],s_{1}\right)$	$E\left({\hat{u}}_{3}\right)=0.0153$	
	${\hat{u}}_{4}=\left(\left[0.0409,0.0690,0.1028,0.1415\right],s_{2}\right)$	$E\left({\hat{u}}_{4}\right)=0.0295$	

**Table 9:** Comparison of the TF2DLWPBM operator with other aggregation operators.

Methods	Aggregation Operator/Method	Whether Captures the Interrelationship among the Attributes	Ranking
Li [21]	TF2DLPGWA	No	$X_{4}≻X_{1}≻X_{3}≻X_{2}$
Dutta [29]	LW-2TLPBM	Yes	$X_{4}≻X_{1}≻X_{3}≻X_{2}$
Shi [49]	TTFLWBM	No	$X_{4}≻X_{1}≻X_{3}≻X_{2}$
Liu [18]	Topsis	No	$X_{4}≻X_{3}≻X_{1}≻X_{2}$
Proposed method	TF2DLWPBM	Yes	$X_{4}≻X_{1}≻X_{3}≻X_{2}$

## References

[1] Xu Z. (2004). A method based on linguistic aggregation operators for group decision making with linguistic preference relations. Inf. Sci..

[2] Cheng P.F., Zhou X.H., Tang X.P. (2006). A decision making method based on the uncertain linguistic. Stat. Decis..

[3] Zhang N.C., Li Z.L., Liu C.Y. (2007). Choice of landing area based on uncertain linguistic information multiple attribute decision making. Ship Electron. Eng..

[4] Xu Z.S., Bustince H., Herrera F., Montero J. (2008). Linguistic aggregation operators: An overview. Fuzzy Sets and Extensions: Representation, Aggregation and Models.

[5] Liu F., Zhang W.G., Wang Z.X. (2012). A goal programming model for incomplete interval multiplicative preference relations and its application in group decision-making. Eur. J. Oper. Res..

[6] Chen T.-Y., Chang C.-H., Lu J.R. (2013). The extended qualiflex method for multiple criteria decision analysis based on interval type-2 fuzzy sets and applications to medical decision making. Eur. J. Oper. Res..

[7] Liu P., Jin F. (2012). Methods for aggregating intuitionistic uncertain linguistic variables and their application to group decision making. Inf. Sci..

[8] Zadeh L.A. (1965). Fuzzy sets. Inf. Control.

[9] Zadeh L.A. (1975). The concept of a linguistic variable and its application to approximate reasoning—I. Inf. Sci..

[10] Zadeh L.A. (1975). The concept of a linguistic variable and its application to approximate reasoning—II. Inf. Sci..

[11] Zadeh L.A. (1975). The concept of a linguistic variable and its application to approximate reasoning—III. Inf. Sci..

[12] Wei G. (2010). Some induced geometric aggregation operators with intuitionistic fuzzy information and their application to group decision making. Appl. Soft Comput..

[13] Li D.-F. (2011). The gowa operator based approach to multiattribute decision making using intuitionistic fuzzy sets. Math. Comput. Model..

[14] Dong J., Wan S. (2016). A new method for multi-attribute group decision making with triangular intuitionistic fuzzy numbers. Kybernetes.

[15] Biswas P., Pramanik S., Giri B.C. (2015). Topsis method for multi-attribute group decision-making under single-valued neutrosophic environment. Neural Comput. Appl..

[16] Wang J., Wang J.Q., Zhang H.Y., Chen X.H. (2016). Multi-criteria group decision-making approach based on 2-tuple linguistic aggregation operators with multi-hesitant fuzzy linguistic information. Int. J. Fuzzy Syst..

[17] Zhu W.D., Zhou G.Z., Yang S.L. (2009). An approach to group decision making based on 2-dimensional linguistic assessment information. Syst. Eng..

[18] Liu P.D., Zhang X. (2012). An approach to group decision making based on 2-dimensional uncertain linguistic assessment information. Technol. Econ. Dev. Econ..

[19] Zhang C., Zhou G.Z., Zhu W.D. (2012). Research on peer review system for the National Science Foundation based on two-dimensionalal semantics evidence reasoning. China Soft Sci..

[20] Yu X.H., Xu Z.S., Liu S.S., Chen Q. (2010). 2012, Multi-criteria decision making with 2-dimensional linguistic aggregation techniques. Int. J. Intell. Syst..

[21] Li Y., Wang Y., Liu P. (2015). Multiple attribute group decision-making methods based on trapezoidal fuzzy two-dimension linguistic power generalized aggregation operators. Soft Comput..

[22] Liu P.D., Wang Y.M. (2015). The aggregation operators based on the 2-dimension uncertain linguistic information and their application to decision making. Int. J. Mach. Learn. Cybern..

[23] Liu P., Teng F. (2016). Multiple attribute decision-making method based on 2-dimension uncertain linguistic density generalized hybrid weighted averaging operator. Soft Comput..

[24] Liu P., He L., Yu X. (2015). Generalized hybrid aggregation operators based on the 2-dimension uncertain linguistic information for multiple attribute group decision making. Group Decis. Negot..

[25] Bonferroni C. (1950). Sulle medie multiple di potenze. Boll. Mat. Ital..

[26] Yager R.R. (2009). On generalized Bonferroni mean operators for multi-criteria aggregation. Int. J. Approx. Reason..

[27] Yager R.R., Beliakov G., James S. On generalized Bonferroni means. Proceedings of the Eurofuse Workshop Preference Modelling Decision Analysis.

[28] Beliakov G., James S., Yager R.R. (2010). Generalized Bonferroni mean operators in multi-criteria aggregation. Fuzzy Sets Syst..

[29] Dutta B., Guha D. (2015). Partitioned bonferroni mean based on linguistic 2-tuple for dealing with multi-attribute group decision making. Appl. Soft Comput..

[30] Wei G., Zhao X., Lin R., Wang H. (2013). Corrigendum to uncertain linguistic bonferroni mean operators and their application to multiple attribute decision making. Appl. Math. Model..

[31] Xia M., Xu Z.S., Zhu B. (2012). Generalized intuitionistic fuzzy Bonferroni means. Int. J. Intell. Syst..

[32] Xu Z.S., Yager R.R. (2011). Intuitionistic fuzzy Bonferroni means. IEEE Trans. Syst. Man Cybern. B.

[33] Jiang X.P., Wei G.W. (2014). Some Bonferroni mean operators with 2-tuple linguistic information and their application to multiple attribute decision making. J. Intell. Fuzzy Syst..

[34] Liu X., Tao Z., Chen H., Zhou L. (2016). A new interval-valued 2-tuple linguistic bonferroni mean operator and its application to multiattribute group decision making. Int. J. Fuzzy Syst..

[35] Liu P.D., Chen S.M., Liu J.L. (2017). Multiple attribute group decision making based on intuitionistic fuzzy interaction partitioned Bonferroni mean operators. Inf. Sci..

[36] Liu Z.M., Liu P.D. (2017). Intuitionistic uncertain linguistic partitioned Bonferroni means and their application to multiple attribute decision-making. Int. J. Syst. Sci..

[37] Li R.J. (2002). Theory and Application on the Fuzzy Multiple Attribute Decision Making.

[38] Su Z.B. (2006). Study on Consistency Issues and Sorting Methods Based on Three Kinds of Judgement Matrix in FAHP.

[39] Degani R., Bortolan G. (1988). The problem of linguistic approximation in clinical decision making. Int. J. Approx. Reason..

[40] Herrera F., Herrera-Viedma E., Verdegay J.L. (1996). A model of consensus in group decision making under linguistic assessments. Fuzzy Sets Syst..

[41] Herrera F., Herrera-Viedma E. (2000). Linguistic decision analysis: Steps for solving decision problems under linguistic information. Fuzzy Sets Syst..

[42] Dutta B., Guha D. Trapezoidal intuitionistic fuzzy Bonferroni means and its application in multi-attribute decision making. Proceedings of the IEEE International Conference on Fuzzy System (FUZZ-IEEE, 2013).

[43] Copping A., Hanna L., Van Cleve B., Blake K., Anderson R.M. (2015). Environmental risk evaluation system—An approach to ranking risk of ocean energy development on coastal and estuarine environments. Estuaries Coasts.

[44] Roussiez V., Ludwig W., Radakovitch O., Probst J.-L., Monaco A., Charrière B., Buscail R. (2011). Fate of metals in coastal sediments of a mediterranean flood-dominated system: An approach based on total and labile fractions. Estuar. Coast. Shelf Sci..

[45] Li X., Hipel K.W., Dang Y. (2015). An improved grey relational analysis approach for panel data clustering. Expert. Syst. Appl..

[46] Yin K., Zhang Y., Li X. (2017). Research on storm-tide disaster losses in China using a new grey relational analysis model with the dispersion of panel data. Int. J. Environ. Res. Public Health.

[47] Polidoro B.A., Carpenter K.E., Collins L. (2010). The Loss of Species: Mangrove Extinction Risk and Geographic Areas of Global Concern. PLoS ONE.

[48] Liu P.D. (2009). A novel method for hybrid multiple attribute decision making. Knol.-Based Syst. Sci..

[49] Shi L.L. (2016). The Research on Aggregation Operators Based on Trapezoidal Two-Dimension Linguistic Numbers. Master’s Thesis.

